# Prediction of Moderate-to-Severe Sepsis-Associated Acute Kidney Injury Using a Dual-Timepoint Machine Learning Model: Development, Multiregional Validation, and Clinical Deployment Study

**DOI:** 10.2196/73840

**Published:** 2025-09-30

**Authors:** Xinbo Ge, Weiwei Chen, Jianshan Shi, Jiaqiang Zhang, Hao Tai, Ying Zhang, Biao Wang, Wei Liu, Song Chen, Huirui Han

**Affiliations:** 1 Department of Critical Care Medicine The First Affiliated Hospital of Hainan Medical University Haikou China; 2 Emergency and Trauma College Hainan Medical University Haikou China; 3 School of Intelligent Medicine and Technology Hainan Engineering Research Center for Health Big Data Hainan Medical University Haikou China; 4 Department of Interventional Vascular Surgery The First Affiliated Hospital of Hainan Medical University Haikou China; 5 Department of Radiology Wanning Hospital The First Affiliated Hospital of Hainan Medical University Wanning China; 6 Department of Emergency Medicine Wanning Hospital The First Affiliated Hospital of Hainan Medical University Wanning China

**Keywords:** acute kidney injury, sepsis, machine learning, predictive model, multicenter validation, SHAP, Shapley additive explanation, clinical decision support

## Abstract

**Background:**

Sepsis-associated acute kidney injury (SA-AKI) is a frequent and life-threatening complication in patients in the intensive care unit (ICU), significantly increasing both mortality rates and the risk of chronic kidney dysfunction. However, existing prediction models have often focused on overall risk and lack severity-based stratification, which limits their clinical applicability.

**Objective:**

This study aimed to identify critical time points in SA-AKI progression development and validate dynamic, stratified machine learning prediction models for moderate-to-severe (Kidney Disease: Improving Global Outcomes guideline stages 2-3) SA-AKI through multicenter, multiregional external validation, ultimately deploying them as publicly accessible, interpretable clinical decision support tools.

**Methods:**

This study used three independent ICU databases: Medical Information Mart for Intensive Care-IV v3.0 (n=12,842; model development and internal validation), electronic ICU collaborative research database v2.0 (n=15,767; North American multicenter external validation), and the First Affiliated Hospital of Hainan Medical University ICU (n=210; Chinese single-center external validation). We identified 48 hours (acute phase) and 7 days (subacute phase) as critical time points. Based on clinical data from the first 24 hours of ICU admission, we used a two-stage feature selection process combining light gradient boosting machine (LightGBM) and Shapley additive explanation (SHAP) cross-validation analysis with clinical expert review, followed by modeling using 8 machine learning algorithms. The optimal model was selected based on the area under the receiver operating characteristic curve (AUC), calibration curves, and decision curve analysis. Internal validation used 5-fold cross-validation, while external validation and subgroup analyses assessed generalizability across different regions and populations. SHAP values and partial dependence plots were used to interpret the influence of key features on predictions.

**Results:**

Our dual-timepoint LightGBM model demonstrated robust predictive performance. For the 48-hour prediction task, the model achieved an AUC of 0.839 (95% CI 0.824-0.854) in the internal test set, with AUCs of 0.770 (95% CI 0.762-0.779) and 0.793 (95% CI 0.726-0.856) in the external validation cohorts, respectively. For the 7-day prediction task, the corresponding AUCs across the three cohorts were 0.834 (95% CI 0.818-0.850), 0.720 (95% CI 0.711-0.729), and 0.773 (95% CI 0.687-0.851), respectively. Subgroup analyses confirmed robust model performance across different age, gender, and comorbidity subgroups. SHAP analysis identified urine output, mechanical ventilation, Sequential Organ Failure Assessment score, creatinine, Glasgow Coma Scale score, and nephrotoxic drug use as core predictive features. Decision curve analysis confirmed that LightGBM provided consistent clinical benefit across different threshold ranges. The optimal LightGBM model was deployed as a publicly accessible web-based prediction app with integrated SHAP interpretability.

**Conclusions:**

This study developed and validated a dynamic, stratified prediction system that provides stage-specific risk assessment for moderate-to-severe SA-AKI. The system underwent rigorous multiregional, multicenter validation and was translated into an interpretable clinical decision support tool, providing a scientific foundation for precision management.

## Introduction

Sepsis-associated acute kidney injury (SA-AKI) is a frequent and high-risk complication among patients in the intensive care unit (ICU), with incidence rates ranging from 40% to 57% in those diagnosed with acute kidney injury (AKI). SA-AKI significantly increases the risk of both in-hospital mortality (from 20% to 40%-60%) and long-term renal dysfunction [1-5]. Current clinical diagnostic methods predominantly rely on serum creatinine (SCr) levels and urine output, as outlined in the 2012 Kidney Disease: Improving Global Outcomes (KDIGO) guidelines. However, these biomarkers are delayed indicators of kidney injury, often resulting in late diagnoses and missed opportunities for timely therapeutic intervention [6-8]. In recent years, machine learning has emerged as a powerful tool for early AKI prediction because of its ability to handle multidimensional, nonlinear data structures [9-13]. For example, Dong et al [14] developed a machine learning–based model capable of predicting AKI in children up to 48 hours earlier than the current diagnostic criteria. Nevertheless, many existing models have limitations such as small sample sizes, a lack of external validation, and a narrow focus on specific populations, all of which restrict their generalizability and clinical utility [15,16]. Ohnuma et al [17] further highlighted these limitations in a multicenter validation study of AKI risk scores across 14 Japanese ICUs, where predictive performance and calibration were found to be suboptimal in new datasets.

For patients at low risk of AKI, unnecessary interventions can disrupt established treatment plans, increase the risk of complications such as infections or trauma, and lead to inefficient use of health care resources [18,19]. Conversely, early intervention in patients at high risk of severe AKI has been demonstrated to yield significant therapeutic benefits [4,6]. The principle of “ineffectiveness equals harm” holds particular importance in critical care, emphasizing that any treatment that fails to deliver its intended effect may have unintended adverse consequences. This underscores the need not only for the accurate prediction of AKI onset but also for stratified assessments of kidney injury severity to optimize treatment strategies. However, most research has focused on predicting the overall risk of AKI, with little attention given to detailed severity stratification [20-22]. In our prior work [23], we developed an SA-AKI prediction model that partially mitigated the diagnostic delays associated with traditional approaches. Nonetheless, the limited ability of the model to stratify kidney injury severity failed to meet clinicians’ needs in practice, thus restricting its broader clinical adoption.

**Figure 1:**
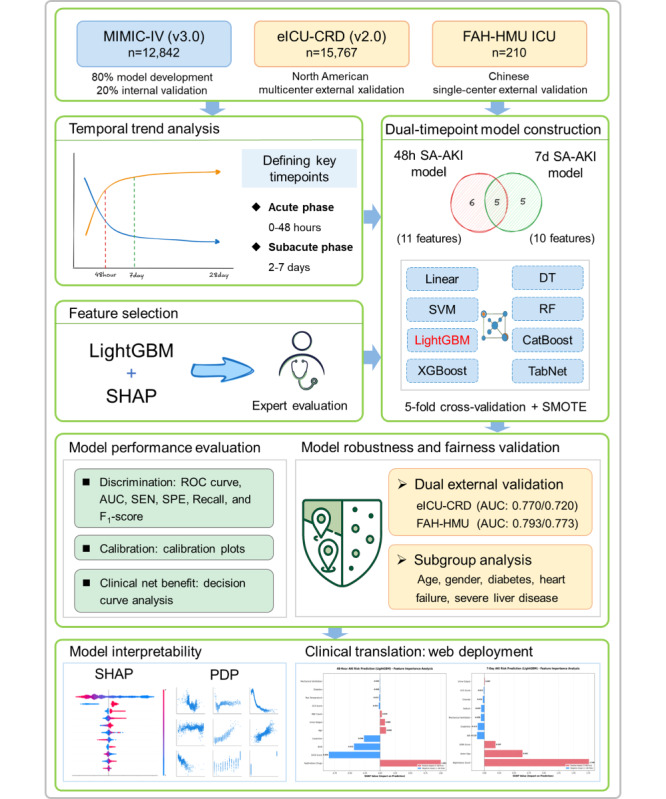
Schematic representation of the dual-timepoint SA-AKI prediction model development and validation workflow. AUC: area under the receiver operating characteristic curve; DT: decision tree; eICU-CRD: electronic intensive care unit collaborative research database; FAH-HMU: First Affiliated Hospital of Hainan Medical University; ICU: intensive care unit; LightGBM: light gradient boosting machine; MIMIC-IV: Medical Information Mart for Intensive Care-IV; PDP: partial dependence plot; RF: random forest; ROC: receiver operating characteristic; SA-AKI: sepsis-associated acute kidney injury; SEN: sensitivity; SHAP: Shapley additive explanations; SPE: Specificity; SVM: support vector machine. XGBoost: extreme gradient boosting.

To address these gaps, this study drew on data from three independent databases: the Medical Information Mart for Intensive Care (MIMIC-IV) database, the electronic ICU collaborative research database (eICU-CRD), and the ICU database of the First Affiliated Hospital of Hainan Medical University (FAH-HMU ICU). We conducted a comprehensive analysis of SA-AKI progression at multiple time points (48 h, 72 h, 7 d, and 28 d). The critical phases of kidney injury progression were identified and used to construct a stratified prediction model tailored to moderate-to-severe SA-AKI. By incorporating Shapley additive explanation (SHAP) values to improve model transparency and interpretability, we aimed to elucidate the contribution of key predictive features to SA-AKI risk, thereby providing robust data-driven support for early clinical intervention strategies (Figure 1).

## Methods

### Data Sources and Patient Cohorts

This study used data from three large, independent databases: the MIMIC-IV v3.0 database, the eICU-CRD v2.0, and FAH-HMU ICU databases. The MIMIC-IV database, developed by the Massachusetts Institute of Technology, contains the deidentified electronic health records of over 50,000 patients admitted to Beth Israel Deaconess Medical Center from 2008 to 2022. The database encompasses detailed information on patient demographics, physiological measurements, laboratory results, diagnoses, and therapeutic interventions [24]. The MIMIC-IV database served as the primary dataset for model development and internal validation. To evaluate the generalizability of the predictive models across multiple centers and different regions, the eICU-CRD and FAH-HMU ICU databases were used for external validation. The eICU-CRD is a multicenter, deidentified database comprising data from over 200,000 patients in ICUs across 208 hospitals collected between 2014 and 2015, providing a diverse patient population with strong heterogeneity [25], which was used to assess the model’s applicability across different medical institutions. The FAH-HMU ICU database collected data on 294 patients with sepsis between 2021 and 2024 (n=210 after screening), which was designed to evaluate the model’s applicability in Chinese patient populations and further validate its predictive performance and clinical utility.

**Figure 2:**
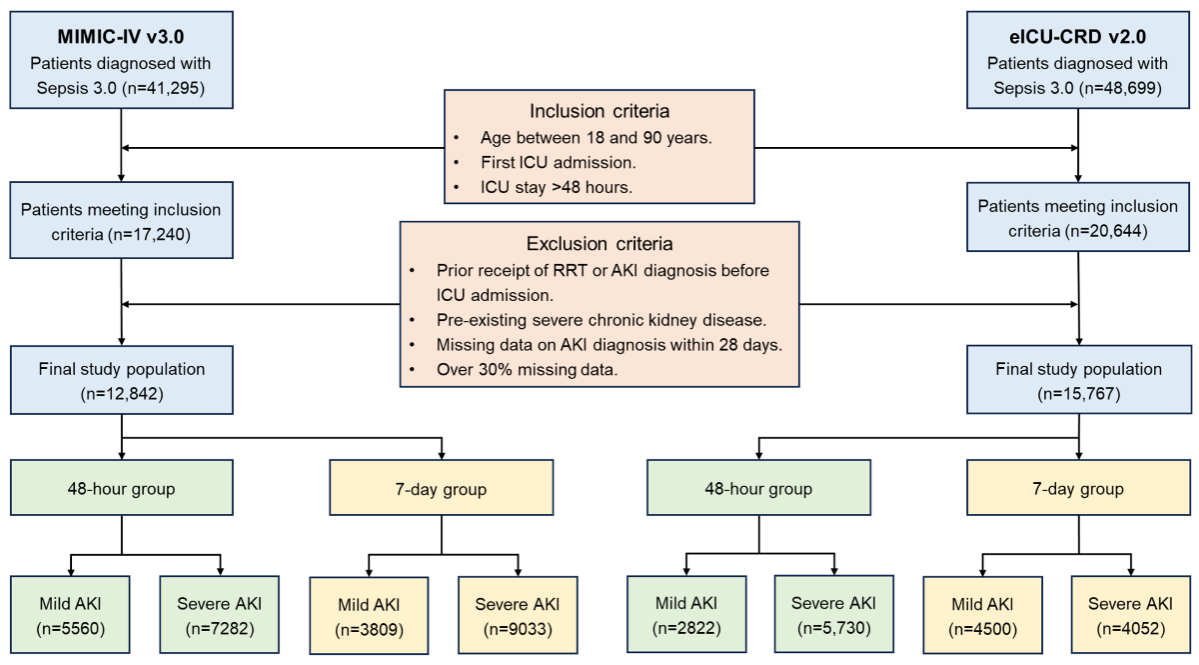
Flowchart for the selection of patients with sepsis with SA-AKI from MIMIC-IV and eICU-CRD databases. AKI: acute kidney injury; eICU-CRD: electronic intensive care unit collaborative research database; ICU: intensive care unit; MIMIC-IV: Medical Information Mart for Intensive Care-IV; RRT: renal replacement therapy; SA-AKI: sepsis-associated acute kidney injury.

The inclusion criteria were as follows: patients aged 18 to 90 years, patients who met the Sepsis 3.0 diagnostic criteria, patients experiencing their first ICU admission, and patients with a stay in the ICU of more than 48 hours. Patients were excluded if they had a prior diagnosis of chronic kidney disease, had received renal replacement therapy, were diagnosed with AKI before ICU admission, or had missing AKI-related data within 28 days or more than 30% missing data overall. Figure 2 and Multimedia Appendix 1 provide detailed illustrations of the data inclusion and exclusion workflows, while Multimedia Appendix 2 comprehensively presents the data preprocessing procedures, the division of training and validation cohorts, and the overall model development and validation process.

**Figure 3:**
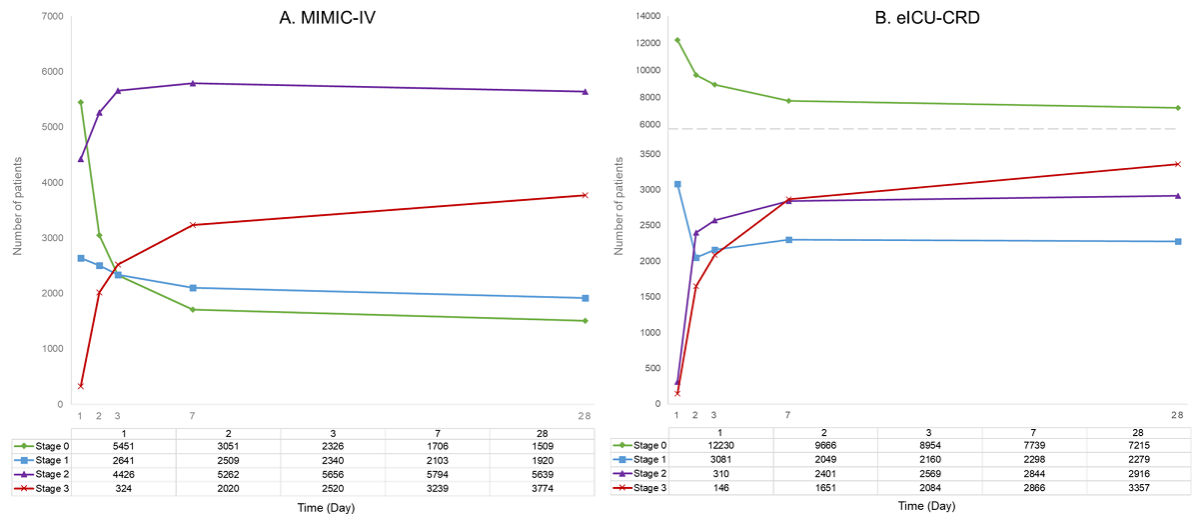
Temporal dynamics of SA-AKI stage distribution in (A) MIMIC-IV and (B) eICU-CRD databases. eICU-CRD: electronic intensive care unit collaborative research database; MIMIC-IV: Medical Information Mart for Intensive Care-IV; SA-AKI: sepsis-associated acute kidney injury.

To assess the dynamic progression of AKI, kidney injury staging was monitored on days 1 (24 h), 2 (48 h), 3, 7, and 28, as shown in Table 1 and Figure 3. Trends in the distribution of patients across different AKI stages (stages 0, 1, 2, and 3) were analyzed. Paired *t* tests were used to compare patient counts between consecutive time points, whereas chi-square tests were used to evaluate the significance of changes in the proportion of patients with severe AKI (stages 2/3). Growth rates were quantified by calculating the percentage change in the number of patients. On the basis of these analyses, 48 hours and 7 days were identified as key prediction time points, reflecting rapid deterioration in the acute phase and cumulative disease progression in the subacute phase. In accordance with the KDIGO guidelines, patients with SA-AKI were stratified into potential-to-mild AKI (stages 0/1) and moderate-to-severe AKI (stages 2/3) groups. The dynamic trends and statistical results are presented in the Results section, with the staging criteria detailed in Multimedia Appendix 3.

**Table 1:** Temporal dynamics of SA-AKI^a^ stage progression and severe AKI^b^ patient distribution in critically ill patients from the MIMIC-IV^c^ database.

Time interval (day)	Stage 0 decrease, n (%)	Stage 1 decrease, n (%)	Stage 2 increase, n (%)	Stage 3 increase, n (%)	Severe AKI^d^ (stage 2-3) growth rate (%)	*P* value^e^
1→2	–2400 (44)	–132 (5)	+836 (18.9)	+1696 (523.5)	46.70	<.001
2→3	–725 (23.8)	–169 (6.7)	+394 (7.5)	+500 (24.8)	16.20	.04
3→7	–620 (26.7)	–237 (10.1)	+138 (2.4)	+719 (28.5)	10.60	.03
2→7	–1345 (44)	–406 (16.2)	+532 (10.1)	+1219 (60.3)	24.70	.02
7→28	–197 (11.5)	–183 (8.7)	–155 (–2.7)	+535 (16.5)	3.90	.08

^a^SA-AKI: sepsis-associated acute kidney injury.

^b^AKI: acute kidney injury.

^c^MIMIC-IV: Medical Information Mart for Intensive Care-IV.

^d^Severe AKI: defined as AKI stage 2-3 according to Kidney Disease: Improving Global Outcomes criteria.

^e^*P* value from the chi-square test, assessing the change in the proportion of severe AKI (stages 2-3).

### Data Extraction

Data management for this study was conducted using Navicat Premium (version 16.2.5; PremiumSoft CyberTech Ltd), with data extracted from the MIMIC-IV database and eICU-CRD using PostgreSQL queries. Data for the FAH-HMU ICU cohort were provided by professional staff from the hospital’s Health Information Management department, who performed the extraction, followed by an institutional review and deidentification process. A total of 59 variables were extracted on the basis of consistent selection criteria, including patient demographics, laboratory test results, therapeutic interventions, comorbidities, and the use of vasoactive medications. For nephrotoxic drugs, data on 19 commonly administered medications were compiled and integrated into a composite nephrotoxicity score. Additionally, the Sequential Organ Failure Assessment (SOFA) score was calculated as a measure of disease severity to provide a comprehensive evaluation of the patients’ clinical conditions. All variables were collected from records within the first 24 hours of ICU admission to ensure temporal alignment and clinical relevance. Detailed variable descriptions are provided in Table 2, and the classification and use of nephrotoxic medications are outlined in Multimedia Appendix 4.

**Table 2:** Key baseline characteristics of patients with SA-AKI^a^ by progression to stages 2-3 AKI^b^ within 48 hours and 7 days (MIMIC-IV^c^ database).

Variables		48-hour group				7-day group		
		AKI stages 0/1 (n=5560)	AKI stages 2/3 (n=7282)	*P* value	AKI stages 0/1 (n=3809)		AKI stages 2/3 (n=9033)	*P* value
**Basic information**								
	Sex (male), n (%)	3197 (57.5)	4140 (56.9)	.76	2190 (57.5)		5147 (57.0)	.87
	Age (years), mean (SD)	60.7 (17.0)	64.6 (15.3)	<.001	59.7 (17.3)		64.2 (15.5)	<.001
**Interventions (24 hours), n (%)**								
	Mechanical ventilation	3553 (63.9)	6633 (91.1)	<.001	2063 (54.2)		8123 (89.9)	<.001
	RRT^d^	28 (0.5)	320 (4.4)	<.001	18 (0.5)		330 (3.7)	<.001
**Comorbidities, n (%)**								
	Arrhythmia	1621 (29.2)	2757 (37.9)	<.001	1028 (27)		3350 (37.1)	<.001
	ARDS^e^	2155 (38.8)	3707 (50.9)	<.001	1286 (33.8)		4576 (50.7)	<.001
	Congestive heart failure	1097 (19.7)	1901 (26.1)	<.001	670 (17.6)		2328 (25.8)	<.001
	Diabetes	1207 (21.7)	1993 (27.4)	<.001	777 (20.4)		2423 (26.8)	<.001
	Severe liver disease	228 (4.1)	729 (10)	<.001	125 (3.3)		832 (9.2)	<.001
**Vital signs, mean (SD)**								
	Urine output (24 h; mL)	2510 (1350)	1380 (962)	<.001	2590 (1390)		1560 (1090)	<.001
	Temperature max (°C)	37.7 (0.837)	37.6 (0.848)	<.001	37.7 (0.829)		37.6 (0.850)	<.001
	SBP^f^ min (mm Hg)	89.6 (15.9)	86.6 (15.8)	<.001	89.3 (15.6)		87.4 (16.0)	<.001
**Laboratory tests, mean (SD)**								
	pH	7.36 (0.108)	7.34 (0.117)	<.001	7.36 (0.104)		7.34 (0.115)	<.001
	Sodium (mmol/L)	138 (5.79)	138 (5.92)	.41	138 (5.73)		138 (5.92)	.04
	Chloride (mmol/L)	104 (7.20)	103 (7.17)	<.001	104 (7.19)		103 (7.17)	<.001
	Lactate (mmol/L)	2.25 (1.84)	2.64 (2.64)	<.001	2.16 (1.72)		2.59 (2.53)	<.001
	RBC^g^ (10^6^/uL)	3.77 (0.842)	3.78 (0.878)	.87	3.75 (0.847)		3.78 (0.870)	.19
	BUN^h^ (mg/dL)	22.4 (19.0)	24.3 (18.9)	<.001	21.9 (19.2)		24.2 (18.8)	<.001
	Creatinine (mg/dL)	1.09 (0.886)	1.25 (1.09)	<.001	1.09 (0.931)		1.22 (1.04)	<.001
	Glucose (mg/dL)	151 (92.9)	158 (91.3)	<.001	149 (97.4)		157 (89.5)	<.001
	Albumin (g/dL)	3.24 (0.715)	3.14 (0.718)	<.001	3.23 (0.710)		3.15 (0.715)	<.001
	INR^i^	1.42 (0.832)	1.56 (1.04)	<.001	1.40 (0.778)		1.54 (1.02)	<.001
	Anion gap (mmol/L)	14.9 (4.72)	15.4 (5.19)	<.001	14.9 (4.71)		15.3 (5.11)	<.001
**Drug use, 24 hours**								
	Nephrotoxic drugs, n (%)	1421 (37.3)	5570 (61.7)	<.001	2399 (43.1)		4592 (63.1)	<.001
	Nephrotoxic score, mean (SD)	0.571 (0.739)	0.850 (0.784)	<.001	0.505 (0.730)		0.824 (0.777)	<.001
**Scores, mean (SD)**								
	SOFA^j^	5.12 (3.02)	6.51 (3.81)	<.001	5.00 (2.90)		6.29 (3.73)	<.001
	GCS^k^	13.1 (3.27)	13.2 (3.30)	<.001	13.1 (3.21)		13.1 (3.33)	.06

^a^SA-AKI: sepsis-associated acute kidney injury.

^b^AKI: acute kidney injury.

^c^MIMIC-IV: Medical Information Mart for Intensive Care-IV.

^d^RRT: renal replacement therapy.

^e^ARDS: acute respiratory distress syndrome.

^f^SBP: systolic blood pressure.

^g^RBC: red blood cell.

^h^BUN: blood urea nitrogen.

^i^INR: international normalized ratio.

^j^SOFA: Sequential Organ Failure Assessment.

^k^GCS: Glasgow Coma Scale.

### Outcome Definitions

The primary outcomes were defined as the stages of AKI at 48 hours and 7 days post-ICU admission. The 2012 KDIGO guidelines were followed for AKI diagnosis and staging, incorporating both SCr and urine output criteria, with the more severe stage between the two parameters recorded as the final classification (Multimedia Appendix 3).

### Model Development and Data Analysis

Data preprocessing and analysis were performed using Python (v3.9.0; Python Software Foundation) and R (v4.4.1; R Core Team). Missing data were imputed using the multivariate imputation by chained equations approach. The dataset was randomly divided into training (80%) and testing (20%) sets, comprising 10,273 and 2569 patients, respectively. The training set was standardized to ensure consistency, and the same transformations were applied to the testing set. To address class imbalance, the synthetic minority oversampling technique was applied to oversample the minority class in the training set. Feature selection was performed using a two-stage workflow: first, through a cross-validation strategy combining light gradient boosting machine (LightGBM) and SHAP values to identify a robust set of core predictive factors; second, these predictors were reviewed by clinical experts based on clinical relevance to finalize the variables for the sepsis-associated kidney injury models.

Eight machine learning algorithms were used for model development: logistic regression, decision tree, random forest, support vector machine, extreme gradient boosting (XGBoost), LightGBM, CatBoost, and TabNet. The MIMIC-IV dataset served as the training and internal validation set, whereas the eICU-CRD and FAH-HMU ICU datasets were used for external validation to evaluate model generalizability. A 5-fold cross-validation was implemented during model training to ensure the stability and reliability of the performance estimates. Model performance was assessed by calculating the area under the receiver operating characteristic curve (AUC). Calibration curves were plotted to evaluate the accuracy of the predicted probabilities, and decision curve analysis (DCA) was performed to assess clinical utility. To validate the rationale of the dual-timepoint independent modeling framework, ablation experiments were conducted on the MIMIC-IV internal test set, comparing the performance of different model architectures. Model performance was evaluated using AUC and *F*_1_-scores as primary metrics (both reported with 95% CIs), with DeLong tests used to compare statistical differences in AUC values between different models. A two-sided *P*<.05 was considered to indicate statistical significance for all tests.

### Subgroup Analysis

To assess model robustness and fairness, a prespecified subgroup analysis was conducted on the MIMIC-IV internal test set and the eICU-CRD external validation cohort. Patients were stratified according to key demographic characteristics (age: <65 vs ≥65 y; gender) and clinical comorbidities (presence of diabetes, congestive heart failure, or severe liver disease [SLD]). In each subgroup, model performance was assessed using the AUC with 95% CIs, calculated via the bootstrap method. Cochran *Q* test and the *I*² statistic were used to test for and quantify heterogeneity of model performance across subgroups, with a *P* value for heterogeneity (*P*het) of <.10 indicating statistical significance. Forest plots were used to visualize all subgroup analysis results, allowing for an intuitive comparison of model performance in each subpopulation relative to the overall population.

### Models Interpretation and Deployment

To enhance model interpretability, SHAP analysis was used to rank feature importance and visualize the marginal contribution of each feature to the model predictions. Partial dependence plots were used to investigate the effects of key features on prediction outcomes. Additionally, a detailed analysis of shared and distinct predictive factors between the 48-hour and 7-day models was conducted to elucidate the key drivers of disease progression across different time windows, providing evidence for early and precise clinical interventions. Finally, we selected the machine learning algorithm model with the best comprehensive performance from the training and validation cohorts for encapsulation and deployed it as a publicly accessible, web-based prediction app to facilitate rapid patient risk assessment by clinical practitioners in real-world scenarios.

### Ethical Considerations

This study was conducted in strict accordance with the “Guidelines for the Development and Reporting of Machine Learning Predictive Models in Biomedical Research.” Data were sourced from three retrospective, deidentified databases: MIMIC-IV v3.0, eICU-CRD v2.0, and the ICU database of the FAH-HMU. The study protocol was reviewed and approved by the Ethics Committee of the FAH-HMU (2025-KYL-148), which granted a waiver for individual patient informed consent owing to the retrospective and anonymized nature of the data. The research team was certified to use the MIMIC-IV and eICU-CRD databases through PhysioNet (Certification No: 58049262) and strictly adhered to the corresponding data use agreements. The establishment of both public databases was also approved by their respective institutional review boards.

## Results

### Temporal Trend Analysis

Based on data from 12,842 patients included in the MIMIC-IV database, we analyzed changes in AKI staging among patients with sepsis across multiple time intervals, as shown in Table 1 and Figure 3A. The analysis revealed an overall trend of a significant decrease in the number of patients with potential-to-mild AKI (stages 0/1) over time, accompanied by a progressive increase in the number of patients with moderate-to-severe AKI (stages 2/3). The most substantial shifts occurred during the acute phase (days 1-2) and the subacute phase (days 2-7). These trends were further validated using data from the eICU-CRD database, which revealed similar distribution patterns, as depicted in Figure 3B.

The acute phase (days 1-2) represented the most pronounced stage of disease progression. The number of Stage 0 patients decreased markedly by 2400 (44% decrease; *P*<.01), whereas the number of Stage 1 patients decreased by 132 (5% reduction; *P*<.05). Simultaneously, the number of patients with moderate-to-severe AKI (stages 2/3) increased significantly, with Stage 3 patients showing the most dramatic increase—an increase of 1696 (523.5% increase; *P*<.01)—representing the largest growth rate observed across all time intervals. Overall, the total growth rate of severe patients during this phase reached 46.7% (*P*<.01), suggesting that this stage may represent a critical window for rapid disease deterioration.

During the subacute phase (days 2-7), the pace of disease deterioration slowed, but severe patient numbers continued to rise steadily. The number of Stage 0 patients decreased by 1345 (44% decrease; *P*<.01), and the number of Stage 1 patients decreased by 406 (16.2% decrease; *P*=.02). In contrast, the number of Stage 2 and Stage 3 patients increased by 532 (10.1% increase; *P*=.02) and 1219 (60.3% increase; *P*<.01), respectively. The total growth rate of severe patients during this phase was 24.7% (*P*=.02), indicating that this stage remains a critical period for clinical intervention.

Upon entering the relatively stable phase (days 7-28), AKI staging changes in patients with sepsis slowed significantly. The number of Stage 0 and Stage 1 patients decreased by 197 (11.5% decrease; *P*=.08) and 183 (8.7% decrease; *P*=.08), respectively. The number of Stage 2 patients also decreased slightly (a decrease of 155, –2.7%), whereas the number of Stage 3 patients showed markedly reduced growth, only increasing by 535 (16.5% increase; *P*=.08). The overall growth rate of severe patients decreased to 3.9%, suggesting that disease progression tends to stabilize during this stage, with reduced urgency for intervention.

Comprehensive analysis revealed that days 1-2 and days 2-7 represent the two critical stages for SA-AKI disease progression. The days 1-2 stage marks rapid deterioration in the acute phase, whereas the days 2-7 stage reflects cumulative disease progression effects in the subacute phase. In contrast, kidney injury progression tends to stabilize during the 7-28-day period. The validation results from the eICU-CRD database were highly consistent with the MIMIC-IV data (Figure 3B), further demonstrating the robustness of these findings (detailed patient distribution is provided in Multimedia Appendices 5 and 6). Therefore, we selected 48 hours (day 2) and 7 days as key time points for predictive modeling, providing a scientific foundation for early intervention and risk prediction of SA-AKI.

### Model Features and Selection

#### Baseline Characteristics

The training and internal validation cohorts included 12,873 patients with sepsis from the MIMIC-IV database, whereas the external validation cohorts included 15,767 patients with sepsis from the eICU-CRD database and 210 patients with sepsis from the FAH-HMU ICU. A total of 59 candidate variables were extracted from these datasets, with 19 nephrotoxic drugs consolidated into two variables: whether nephrotoxic drugs were used and a nephrotoxic drug score (Multimedia Appendix 4). Baseline characteristics for the MIMIC-IV cohorts were stratified according to AKI severity (Table 2 and Multimedia Appendix 7).

In this study, compared with the mild group (AKI=0/1), patients in the severe group (AKI=2/3) were significantly older than those with mild AKI (48 h: 64.6 vs 60.7 y, *P*<.001; 7 day: 64.2 vs 59.7 y, *P*<.001). No significant differences in gender distribution were detected between the groups (*P*>.05). The severe AKI group had higher SOFA scores (48 h: 6.51 vs 5.12, *P*<.001; 7 day: 6.29 vs 5.00, *P*<.001) and a significantly higher proportion of patients receiving mechanical ventilation and renal replacement therapy within the first 24 hours of ICU admission (*P*<.001). Additionally, comorbidities such as arrhythmia, heart failure, acute respiratory distress syndrome, liver dysfunction, and diabetes were more common in the severe group (*P*<.001).

Vital sign comparisons showed that patients in the severe group had lower systolic blood pressure (*P*<.001) and significantly reduced urine output (48 h: 1380 mL vs 2510 mL, *P*<.01; 7 day: 1560 mL vs 2590 mL, *P*<.001). Laboratory analyses indicated that patients in the severe group had lower pH, base excess, oxygenation index, albumin levels, and chloride concentrations compared to the mild group (*P*<.01), whereas PaCO2, anion gap, lactate, blood urea nitrogen (BUN), creatinine, bilirubin, prothrombin time, international normalized ratio (INR), and blood glucose levels were significantly higher than those in the mild group (*P*<.01). In terms of medication use, the proportion and scores of nephrotoxic drug use, as well as the rates of vasoactive drug administration, were significantly higher in the severe group than in the mild group (*P*<.01). In the above analyses, the data trends for the 48-hour and 7-day groups were essentially consistent.

#### Feature Selection

To ensure statistical robustness and clinical relevance, feature selection was performed through a two-stage workflow. First, under a stratified cross-validation framework, we implemented a strategy combining LightGBM feature importance and SHAP contribution values. This step identified a set of candidate features that demonstrated consistent importance. Subsequently, this candidate set was submitted to a team of critical care experts for comprehensive review. The experts evaluated the pathophysiological relevance and clinical applicability of each feature based on their clinical experience and existing medical evidence to determine the final variable set for the predictive models. Through this two-stage process, the 48-hour prediction model ultimately selected 11 features, including urine output, mechanical ventilation, SOFA score, and nephrotoxic drug use, among others. The 7-day prediction model selected 10 features, such as creatinine, mechanical ventilation, INR, and nephrotoxic drug score, among others. These finally selected features ranked highly in both importance assessment methods, as detailed in Multimedia Appendix 8. They encompass key dimensions, including renal function status, metabolic disturbances, inflammatory responses, comorbidities, and therapeutic interventions, and formed the foundation for model development input variables.

### Model Performance Comparison

#### Model 1: 48-Hour Moderate-to-Severe Kidney Injury Risk Prediction Model

Within the 48-hour evaluation window, we conducted a systematic comparative analysis of eight machine learning models using training, internal test, and two external validation datasets (Table 3). Gradient boosting decision tree–based algorithms demonstrated relatively superior performance compared to other tested approaches. LightGBM, XGBoost, and CatBoost showed consistent performance advantages across multiple evaluation metrics. Among these three models, LightGBM obtained the highest AUC.

**Table 3:** Performance comparison of six models in predicting the risk of moderate-to-severe SA-AKIa (AKI=Stages 2/3) in the 48-hour group across different datasets.

Model			Accuracy (95% CI)		Precision (95% CI)		Recall (95% CI)		*F*_1_-score (95% CI)		Specificity (95% CI)		AUC^b^ (95% CI)
**Training set (MIMIC-IV^c^)**													
	Linear	0.740 (0.731-0.749)		0.742 (0.733-0.750)		0.740 (0.730-0.749)		0.739 (0.730-0.748)		0.695 (0.679-0.710)		0.830 (0.824-0.838)	
	DT^d^	0.697 (0.676-0.708)		0.697 (0.676-0.708)		0.697 (0.676-0.708)		0.697 (0.676-0.708)		0.695 (0.666-0.708)		0.697 (0.676-0.708)	
	RF^e^	0.767 (0.762-0.774)		0.768 (0.763-0.776)		0.767 (0.762-0.775)		0.766 (0.762-0.774)		0.795 (0.783-0.807)		0.859 (0.853-0.867)	
	SVM^f^	0.742 (0.734-0.748)		0.745 (0.737-0.750)		0.742 (0.733-0.748)		0.742 (0.732-0.747)		0.691 (0.673-0.702)		0.836 (0.832-0.846)	
	XGBoost^g^	0.771 (0.760-0.780)		0.771 (0.761-0.782)		0.770 (0.760-0.781)		0.770 (0.760-0.780)		0.801 (0.782-0.818)		0.860 (0.854-0.869)	
	LightGBM^h^	0.772 (0.765-0.779)		0.773 (0.768-0.780)		0.772 (0.768-0.779)		0.771 (0.768-0.779)		0.799 (0.794-0.808)		0.862 (0.858-0.871)	
	CatBoost^i^	0.772 (0.765-0.777)		0.773 (0.766-0.779)		0.772 (0.765-0.778)		0.771 (0.764-0.777)		0.809 (0.802-0.819)		0.859 (0.851-0.867)	
	TabNet^j^	0.757 (0.752-0.763)		0.758 (0.753-0.767)		0.757 (0.752-0.764)		0.757 (0.751-0.763)		0.792 (0.783-0.817)		0.845 (0.833-0.855)	
**Internal validation (MIMIC-IV)**													
	Linear	0.733 (0.723-0.755)		0.735 (0.717-0.752)		0.734 (0.716-0.751)		0.734 (0.716-0.751)		0.696 (0.667-0.723)		0.819 (0.802-0.836)	
	DT	0.666 (0.647-0.685)		0.662 (0.643-0.680)		0.663 (0.644-0.683)		0.662 (0.643-0.681)		0.641 (0.611-0.670)		0.663 (0.644-0.683)	
	RF	0.744 (0.728-0.761)		0.743 (0.725-0.759)		0.747 (0.730-0.764)		0.743 (0.725-0.759)		0.763 (0.739-0.789)		0.827 (0.811-0.843)	
	SVM	0.735 (0.718-0.751)		0.730 (0.713-0.748)		0.728 (0.711-0.746)		0.729 (0.712-0.746)		0.678 (0.650-0.706)		0.818 (0.801-0.834)	
	XGBoost	0.754 (0.738-0.770)		0.752 (0.736-0.769)		0.757 (0.740-0.773)		0.752 (0.736-0.769)		0.777 (0.750-0.799)		0.838 (0.822-0.853)	
	LightGBM	0.757 (0.740-0.774)		0.754 (0.738-0.770)		0.758 (0.741-0.774)		0.755 (0.738-0.771)		0.764 (0.739-0.788)		0.839 (0.824-0.854)	
	CatBoost	0.749 (0.732-0.765)		0.812 (0.732-0.765)		0.732 (0.701-0.746)		0.765 (0.747-0.782)		0.782 (0.755-0.806)		0.836 (0.819-0.852)	
	TabNet	0.748 (0.732-0.764)		0.799 (0.777-0.820)		0.742 (0.718-0.763)		0.769 (0.752-0.787)		0.756 (0.730-0.781)		0.837 (0.821-0.853)	
**External validation (eICU-CRD** ^k^ **)**													
	Linear	0.695 (0.687-0.702)		0.634 (0.626-0.642)		0.659 (0.650-0.668)		0.639 (0.630-0.648)		0.733 (0.724-0.741)		0.729 (0.720-0.738)	
	DT	0.613 (0.606-0.620)		0.597 (0.591-0.604)		0.626 (0.619-0.635)		0.581 (0.574-0.588)		0.599 (0.591-0.608)		0.626 (0.619-0.635)	
	RF	0.701 (0.694-0.708)		0.662 (0.655-0.668)		0.704 (0.697-0.713)		0.663 (0.655-0.671)		0.698 (0.690-0.706)		0.756 (0.748-0.765)	
	SVM	0.613 (0.606-0.621)		0.582 (0.575-0.589)		0.605 (0.597-0.614)		0.572 (0.564-0.580)		0.662 (0.613-0.631)		0.696 (0.688-0.706)	
	XGBoost	0.701 (0.694-0.708)		0.661 (0.654-0.668)		0.704 (0.695-0.712)		0.663 (0.655-0.670)		0.699 (0.690-0.707)		0.764 (0.756-0.773)	
	LightGBM	0.713 (0.706-0.720)		0.671 (0.664-0.678)		0.715 (0.708-0.724)		0.675 (0.667-0.682)		0.711 (0.703-0.719)		0.770 (0.762-0.779)	
	CatBoost	0.699 (0.692-0.707)		0.444 (0.432-0.457)		0.679 (0.666-0.694)		0.537 (0.526-0.549)		0.706 (0.698-0.715)		0.743 (0.733-0.752)	
	TabNet	0.721 (0.714-0.729)		0.471 (0.458-0.485)		0.693 (0.679-0.707)		0.561 (0.548-0.573)		0.731 (0.723-0.740)		0.769(0.761,0.778)	
**External Validation (FAH-HMU ICU^l^)**													
	Linear	0.676 (0.614-0.738)		0.676 (0.614-0.738)		1.000 (1.000-1.000)		0.807 (0.761-0.849)		0.000 (0.000-0.000)		0.672 (0.591-0.747)	
	DT	0.657 (0.590-0.719)		0.730 (0.654-0.795)		0.782 (0.714-0.849)		0.755 (0.697-0.807)		0.397 (0.279-0.514)		0.589 (0.519-0.657)	
	RF	0.781 (0.729-0.833)		0.782 (0.723-0.840)		0.937 (0.894-0.973)		0.853 (0.810-0.889)		0.456 (0.333-0.568)		0.816 (0.750-0.875)	
	SVM	0.676 (0.614-0.738)		0.676 (0.614-0.738)		1.000 (1.000-1.000)		0.807 (0.761-0.849)		0.000 (0.000-0.000)		0.645 (0.565-0.724)	
	XGBoost	0.733 (0.676-0.790)		0.744 (0.678-0.806)		0.923 (0.875-0.965)		0.824 (0.776-0.865)		0.338 (0.225-0.450)		0.797 (0.732-0.857)	
	LightGBM	0.729 (0.667-0.786)		0.730 (0.665-0.791)		0.951 (0.912-0.986)		0.826 (0.778-0.865)		0.265 (0.161-0.366)		0.793 (0.726-0.856)	
	CatBoost	0.714 (0.652-0.771)		0.725 (0.661-0.785)		0.930 (0.884-0.972)		0.815 (0.767-0.855)		0.265 (0.167-0.365)		0.789 (0.724-0.851)	
	TabNet	0.710 (0.648-0.767)		0.755 (0.692-0.818)		0.845 (0.783-0.902)		0.797 (0.747-0.842)		0.426 (0.311-0.545)		0.769 (0.701-0.832)	

^a^SA-AKI: sepsis-associated acute kidney injury.

^b^AUC: area under the receiver operating characteristic curve.

^c^MIMIC-IV: Medical Information Mart for Intensive Care IV.

^d^DT: decision tree.

^e^RF: random forest.

^f^SVM: support vector machine.

^g^XGBoost: extreme gradient boosting.

^h^LightGBM: light gradient boosting machine.

^i^CatBoost: categorical Boosting.

^j^TabNet: tabular Network.

^k^eICU-CRD: electronic intensive care unit collaborative research database.

^l^FAH-HMU ICU: First Affiliated Hospital of Hainan Medical University intensive care unit.

**Figure 4:**
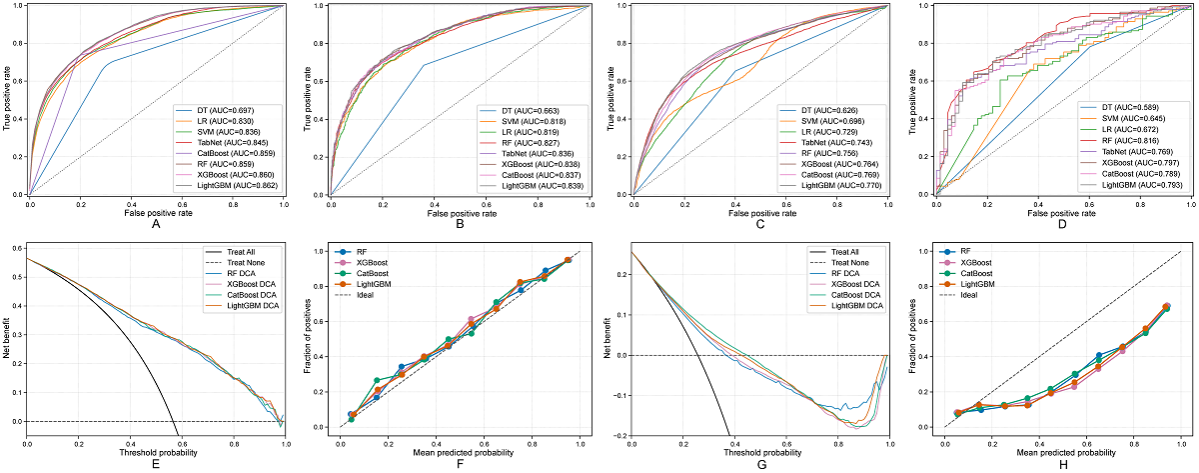
Performance evaluation of machine learning models for 48-hour SA-AKI risk prediction: (A) ROC curves for MIMIC-IV training, (B) ROC curves for MIMIC-IV internal validation, (C) ROC curves for eICU-CRD external validation, (D) ROC curves for FAH-HMU ICU external validation, (E) DCA for MIMIC-IV internal validation, (F) calibration curve for MIMIC-IV internal validation, (G) DCA for eICU-CRD external validation, and (H) calibration curve for eICU-CRD external validation. AUC: area under the curve; CatBoost: categorical boosting; DCA: decision curve analysis; DT: decision tree; eICU-CRD: electronic intensive care unit collaborative research database; FAH-HMU ICU: First Affiliated Hospital of Hainan Medical University intensive care unit; LightGBM: light gradient boosting machine; LR: logistic regression; SVM: support vector machine; RF: random forest; ROC: receiver operating characteristic; SA-AKI: sepsis-associated acute kidney injury; TabNet: tabular network; XGBoost: eXtreme gradient boosting.

Figure 4 systematically demonstrates LightGBM’s discriminative ability across four independent cohorts. In the MIMIC-IV training set, LightGBM achieves an AUC of 0.862 (95% CI 0.858-0.871; Figure 4A). This performance was stably reproduced in the MIMIC-IV internal test set, with an AUC of 0.839 (95% CI 0.824-0.854), confirming internal generalizability (Figure 4B). In cross-system external validation, the model achieved a robust AUC of 0.770 (95% CI 0.762-0.779) in the North American multicenter eICU-CRD cohort (Figure 4C) and achieved similar performance in the Chinese single-center FAH-HMU cohort, with an AUC of 0.793 (95% CI 0.726-0.856; Figure 4D). These results collectively confirmed the model’s strong generalizability across different health care systems and patient populations.

In clinical utility assessment, DCA further established LightGBM’s comprehensive advantages (Figure 4). Whether in the MIMIC-IV internal test set (Figure 4E) or the eICU-CRD external validation cohort (Figure 4G), LightGBM consistently provided higher net benefit across the broadest range of clinically relevant threshold probabilities. In the FAH-HMU ICU external validation (Multimedia Appendix 9), LightGBM also demonstrated good clinical decision-making value, providing positive net benefit within the 0.3-0.8 threshold probability range. Additionally, the model’s calibration curves also showed good consistency, indicating that its predicted probabilities closely matched actual event occurrence rates (Figure 4F,H and Multimedia Appendix 9). In summary, based on its comprehensive advantages in discrimination, generalizability, and clinical net benefit, LightGBM was selected as the final 48-hour prediction model.

#### Model 2: 7-Day Moderate-to-Severe Kidney Injury Risk Prediction Model

**Figure 5:**
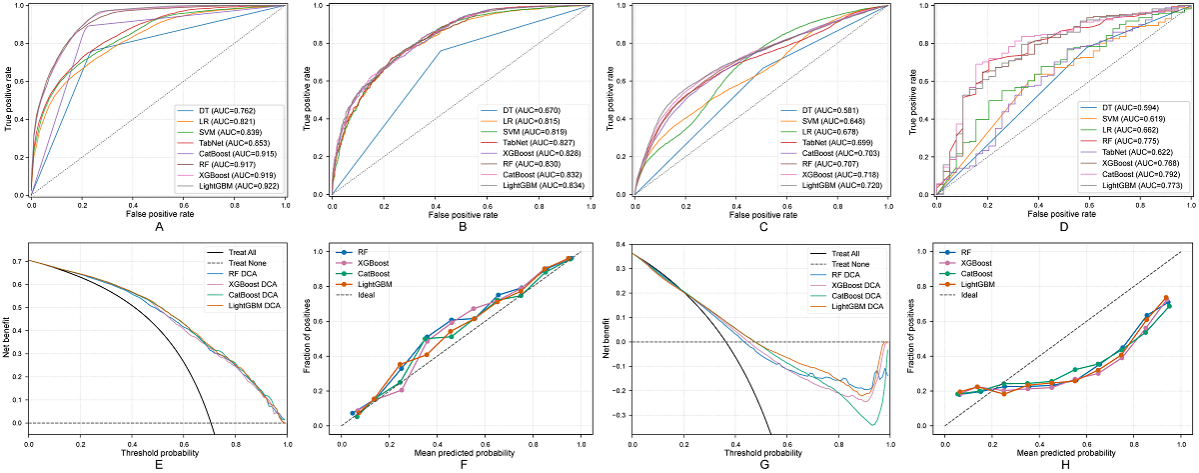
Performance evaluation of machine learning models for 7-day SA-AKI risk prediction: (A) ROC curves for MIMIC-IV training, (B) ROC curves for MIMIC-IV internal validation, (C) ROC curves for eICU-CRD external validation, (D) ROC curves for FAH-HMU ICU external validation, (E) DCA for MIMIC-IV internal validation, (F) calibration curve for MIMIC-IV internal validation, (G) DCA for eICU-CRD external validation, and (H) calibration curve for eICU-CRD external validation. AUC: area under the curve; CatBoost: categorical boosting; DCA: decision curve analysis; DT: decision tree; eICU-CRD: electronic intensive care unit collaborative research database; FAH-HMU ICU: First Affiliated Hospital of Hainan Medical University intensive care unit; LightGBM: light gradient boosting machine; LR: logistic regression; SVM: support vector machine; RF: random forest; ROC: receiver operating characteristic; SA-AKI: sepsis-associated acute kidney injury; TabNet: tabular network; XGBoost: eXtreme gradient boosting.

For this longer prediction window of 7 days, we similarly conducted a systematic evaluation of eight machine learning models (Table 4). In the MIMIC-IV training set, LightGBM again stood out with an AUC of 0.922 (95% CI 0.918-0.927), forming the first-tier performance group together with XGBoost (AUC 0.919) and random forest (AUC 0.917). The model showed consistent performance in internal and external validation (Figure 5): in the MIMIC-IV internal test set (Figure 5B) and eICU-CRD external validation cohort (Figure 5C), LightGBM achieved AUCs of 0.834 and 0.720, respectively; in the FAH-HMU ICU cohort (Figure 5D), the model achieved similar performance (AUC 0.773), with sensitivity of 0.848 and specificity of 0.487.

In clinical utility assessment, LightGBM’s advantages remained clear. DCA in Figure 5 showed that whether in the internal test set (Figure 5E) or external validation sets eICU-CRD and FAH-HMU ICU cohorts (Figure 5G and Multimedia Appendix 9), LightGBM provided higher clinical decision support across broad threshold probability ranges. Combined with its equally excellent model calibration (Figure 5F,H and Multimedia Appendix 9), and considering its overall robustness and clinical utility in multicenter validation, LightGBM was still confirmed as the best model for 7-day prediction tasks.

Based on the above model selection, we further validated the rationale of our dual-timepoint independent modeling framework through ablation experiments. Results showed that the dual independent model achieved AUCs of 0.839 and 0.834 for 48-hour and 7-day prediction tasks, respectively, with no statistically significant differences compared to control model frameworks (DeLong test; all *P*>.05), but demonstrated superior performance in *F*_1_-score stability and balance (Multimedia Appendix 10).

**Table 4:** Performance comparison of the six models in predicting the risk of moderate-to-severe SA-AKI^a^ (AKI=2/3) in the 7-day group across different datasets.

Model		Accuracy (95% CI)	Precision (95% CI)	Recall (95% CI)	*F*_1_-score (95% CI)	Specificity (95% CI)	AUC^b^ (95% CI)
**Training set (MIMIC-IV^c^)**							
	Linear	0.727 (0.723-0.735)	0.730 (0.725-0.739)	0.727 (0.722-0.735)	0.726 (0.722-0.734)	0.668 (0.660-0.676)	0.821 (0.817-0.823)
	DT^d^	0.761 (0.751-0.768)	0.761 (0.751-0.768)	0.761 (0.751-0.768)	0.761 (0.751-0.768)	0.763 (0.757-0.774)	0.761 (0.751-0.768)
	RF^e^	0.839 (0.836-0.843)	0.840 (0.837-0.846)	0.839 (0.836-0.843)	0.838 (0.836-0.843)	0.806 (0.798-0.814)	0.917 (0.914-0.923)
	SVM^f^	0.740 (0.733-0.748)	0.743 (0.738-0.751)	0.740 (0.733-0.748)	0.739 (0.732-0.747)	0.678 (0.660-0.689)	0.839 (0.833-0.841)
	XGBoost^g^	0.846 (0.844-0.847)	0.850 (0.846-0.853)	0.846 (0.844-0.848)	0.845 (0.844-0.847)	0.793 (0.785-0.804)	0.919 (0.915-0.926)
	LightGBM^h^	0.849 (0.844-0.856)	0.857 (0.854-0.863)	0.849 (0.844-0.855)	0.848 (0.843-0.855)	0.774 (0.760-0.787)	0.922 (0.918-0.927)
	CatBoost^i^	0.757 (0.752-0.763)	0.758 (0.753-0.767)	0.757 (0.752-0.764)	0.757 (0.751-0.763)	0.792 (0.783-0.817)	0.845 (0.833-0.855)
	TabNet^j^	0.772 (0.765-0.777)	0.773 (0.766-0.779)	0.772 (0.765-0.778)	0.771 (0.764-0.777)	0.809 (0.851-0.867)	0.859 (0.851-0.867)
**Internal validation (MIMIC-IV 3.0)**							
	Linear	0.751 (0.734-0.768)	0.710 (0.693-0.728)	0.734 (0.715-0.753)	0.718 (0.700-0.736)	0.692 (0.657-0.724)	0.815 (0.797-0.833)
	DT	0.707 (0.688-0.724)	0.657 (0.638-0.676)	0.670 (0.649-0.690)	0.662 (0.642-0.681)	0.579 (0.545-0.614)	0.670 (0.649-0.690)
	RF	0.783 (0.767-0.799)	0.741 (0.721-0.761)	0.713 (0.695-0.733)	0.724 (0.705-0.744)	0.545 (0.510-0.579)	0.830 (0.813-0.846)
	SVM	0.761 (0.743-0.777)	0.719 (0.700-0.743)	0.739 (0.719-0.757)	0.726 (0.707-0.743)	0.684 (0.650-0.717)	0.819 (0.802-0.838)
	XGBoost	0.791 (0.775-0.806)	0.752 (0.732-0.771)	0.721 (0.700-0.740)	0.733 (0.712-0.751)	0.550 (0.513-0.583)	0.828 (0.811-0.844)
	LightGBM	0.803 (0.787-0.818)	0.781 (0.759-0.803)	0.714 (0.695-0.733)	0.734 (0.714-0.754)	0.499 (0.463-0.536)	0.834 (0.818-0.850)
	CatBoost	0.746 (0.729-0.763)	0.709 (0.691-0.726)	0.737 (0.717-0.755)	0.716 (0.697-0.734)	0.714 (0.681-0.746)	0.827 (0.810-0.844)
	TabNet	0.802 (0.788-0.817)	0.780 (0.759-0.799)	0.714 (0.696-0.733)	0.733 (0.714-0.754)	0.500 (0.464-0.535)	0.832 (0.814-0.848)
**External validation (eICU-CRD** ^k^ **)**							
	Linear	0.629 (0.622-0.637)	0.594 (0.586-0.602)	0.591 (0.583-0.598)	0.592 (0.584-0.600)	0.730 (0.722-0.740)	0.678 (0.670-0.687)
	DT	0.557 (0.549-0.565)	0.576 (0.568-0.584)	0.581 (0.573-0.589)	0.555 (0.547-0.563)	0.494 (0.484-0.504)	0.581 (0.573-0.589)
	RF	0.588 (0.581-0.596)	0.624 (0.617-0.631)	0.627 (0.620-0.634)	0.588 (0.580-0.595)	0.483 (0.437-0.449)	0.707 (0.699-0.716)
	SVM	0.544 (0.537-0.553)	0.567 (0.560-0.575)	0.571 (0.563-0.579)	0.543 (0.535-0.551)	0.474 (0.464-0.483）	0.648 (0.639-0.657)
	XGBoost	0.598 (0.590-0.606)	0.631 (0.624-0.638)	0.636 (0.629-0.643)	0.598 (0.590-0.605)	0.498 (0.489-0.508)	0.718 (0.709-0.726)
	LightGBM	0.622 (0.615-0.630)	0.636 (0.629-0.643)	0.645 (0.638-0.653)	0.619 (0.611-0.626)	0.560 (0.550-0.570)	0.720 (0.711-0.729)
	CatBoost	0.593 (0.586-0.601)	0.616 (0.609-0.623)	0.623 (0.616-0.630)	0.592 (0.584-0.599)	0.515 (0.505-0.525)	0.699 (0.691-0.708)
	TabNet	0.621 (0.614-0.629)	0.633 (0.626-0.640)	0.643 (0.635-0.651)	0.618 (0.611-0.626)	0.565 (0.555-0.574)	0.703 (0.694-0.712)
**External validation (FAH-HMU ICU^l^)**							
	Linear	0.814 (0.762-0.862)	0.814 (0.762-0.862)	1.000 (1.000-1.000)	0.898 (0.865-0.926)	0.000 (0.000-0.000)	0.662 (0.564-0.753)
	DT	0.710 (0.652-0.767)	0.853 (0.799-0.908)	0.778 (0.715-0.842)	0.813 (0.766-0.855)	0.410 (0.261-0.581)	0.594 (0.512-0.683)
	RF	0.814 (0.762-0.867)	0.855 (0.801-0.903)	0.930 (0.888-0.965)	0.891 (0.855-0.923)	0.308 (0.162-0.450)	0.775 (0.690-0.857)
	SVM	0.810 (0.757-0.862)	0.813 (0.762-0.862)	0.994 (0.981-1.000)	0.895 (0.862-0.926)	0.000 (0.000-0.000)	0.619 (0.526-0.709)
	XGBoost	0.795 (0.743-0.848)	0.881 (0.832-0.928)	0.865 (0.812-0.914)	0.873 (0.836-0.909)	0.487 (0.317-0.643)	0.768 (0.681-0.851)
	LightGBM	0.781 (0.724-0.838)	0.879 (0.829-0.928)	0.848 (0.796-0.900)	0.863 (0.822-0.901)	0.487 (0.310-0.647)	0.773 (0.687-0.851)
	CatBoost	0.771 (0.714-0.824)	0.918 (0.870-0.959)	0.789 (0.724-0.848)	0.849 (0.804-0.888)	0.692 (0.535-0.833)	0.792 (0.710-0.867)
	TabNet	0.786 (0.729-0.838)	0.832 (0.776-0.881)	0.924 (0.882-0.964)	0.875 (0.836-0.909)	0.179 (0.062-0.303)	0.622 (0.519-0.718)

^a^SA-AKI: sepsis-associated acute kidney injury.

^b^AUC: area under the receiver operating characteristic curve.

^c^MIMIC-IV: Medical Information Mart for Intensive Care-IV.

^d^DT: decision tree.

^e^RF: random forest.

^f^SVM: support vector machine.

^g^XGBoost: extreme gradient boosting.

^h^LightGBM: light gradient boosting machine.

^i^CatBoost: categorical boosting.

^j^TabNet: tabular network.

^k^eICU-CRD: electronic intensive care unit collaborative research database.

^l^FAH-HMU ICU: First Affiliated Hospital of Hainan Medical University intensive care unit.

### Subgroup Analysis

**Figure 6:**
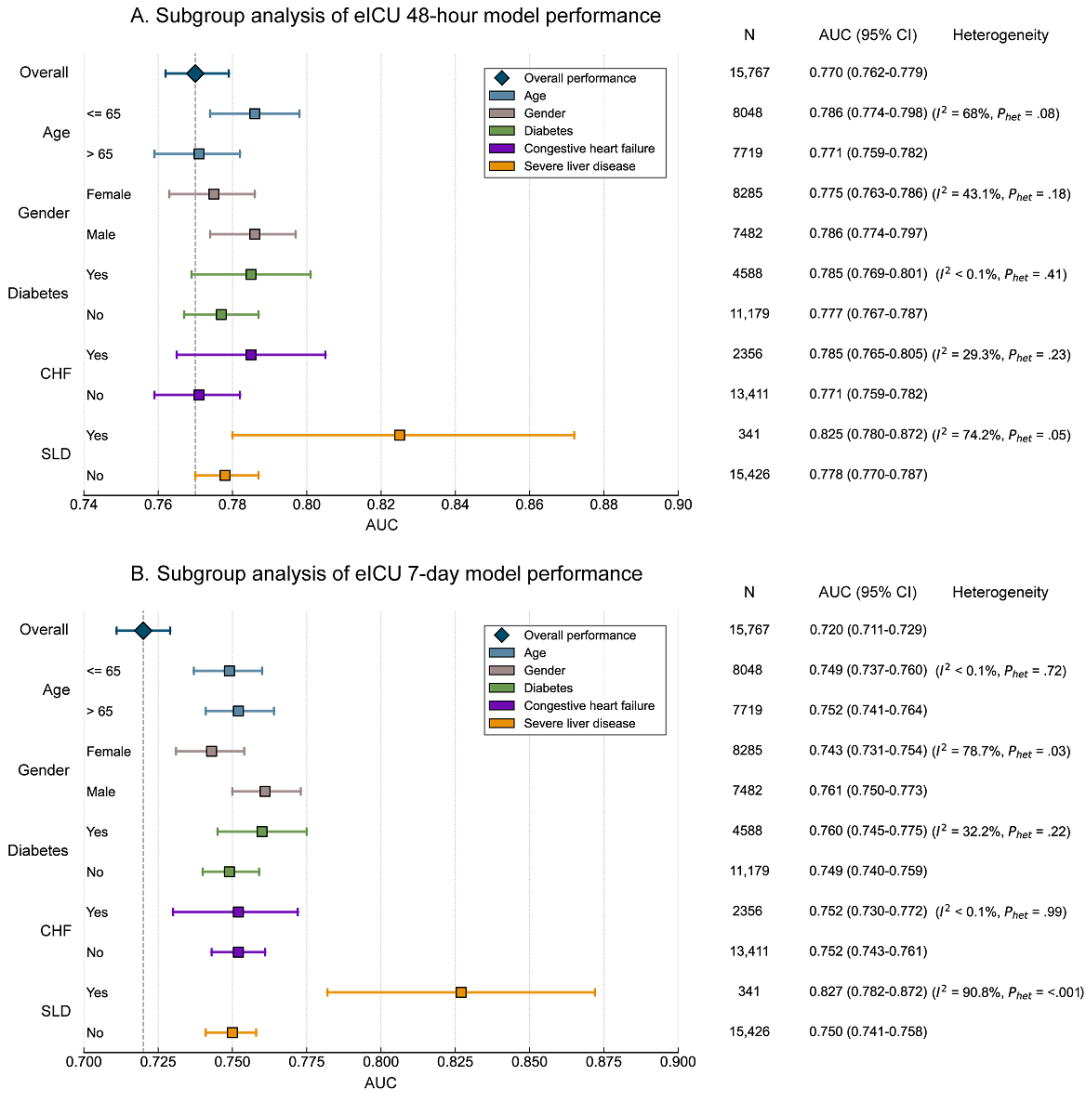
Subgroup analysis of SA-AKI risk prediction model performance in eICU-CRD external validation cohort: (A) 48-hour model performance and (B) 7-day model performance. AUC: area under the receiver operating characteristic curve; CHF: congestive heart failure; eICU: electronic intensive care unit; SA-AKI: sepsis-associated acute kidney injury; SLD: severe liver disease.

To assess model robustness and fairness, we conducted systematic subgroup analyses on the MIMIC-IV internal validation cohort and eICU-CRD external validation cohort. In the eICU-CRD cohort (Figure 6), both 48-hour and 7-day prediction models demonstrated highly consistent and robust performance across most prespecified clinical subgroups. Specifically, in subgroups stratified by age (<65 y vs ≥65 y), gender, receipt of mechanical ventilation, and presence of diabetes or congestive heart failure, model AUC values remained stable compared to the overall population, with nonsignificant heterogeneity (most *P*het≥.10; *I*²≤60%). A notable exception occurred in the subgroup of patients with SLD, where model performance showed significant variation. Although this subgroup had nominally higher AUCs at both time points (0.82-0.83), this result was accompanied by extremely high statistical heterogeneity (*I*²>74%; *P*het≤.05). Similar overall performance consistency was observed in the MIMIC-IV cohort, with detailed results provided in Multimedia Appendices 11 and 12.

### Model Interpretability

**Figure 7:**
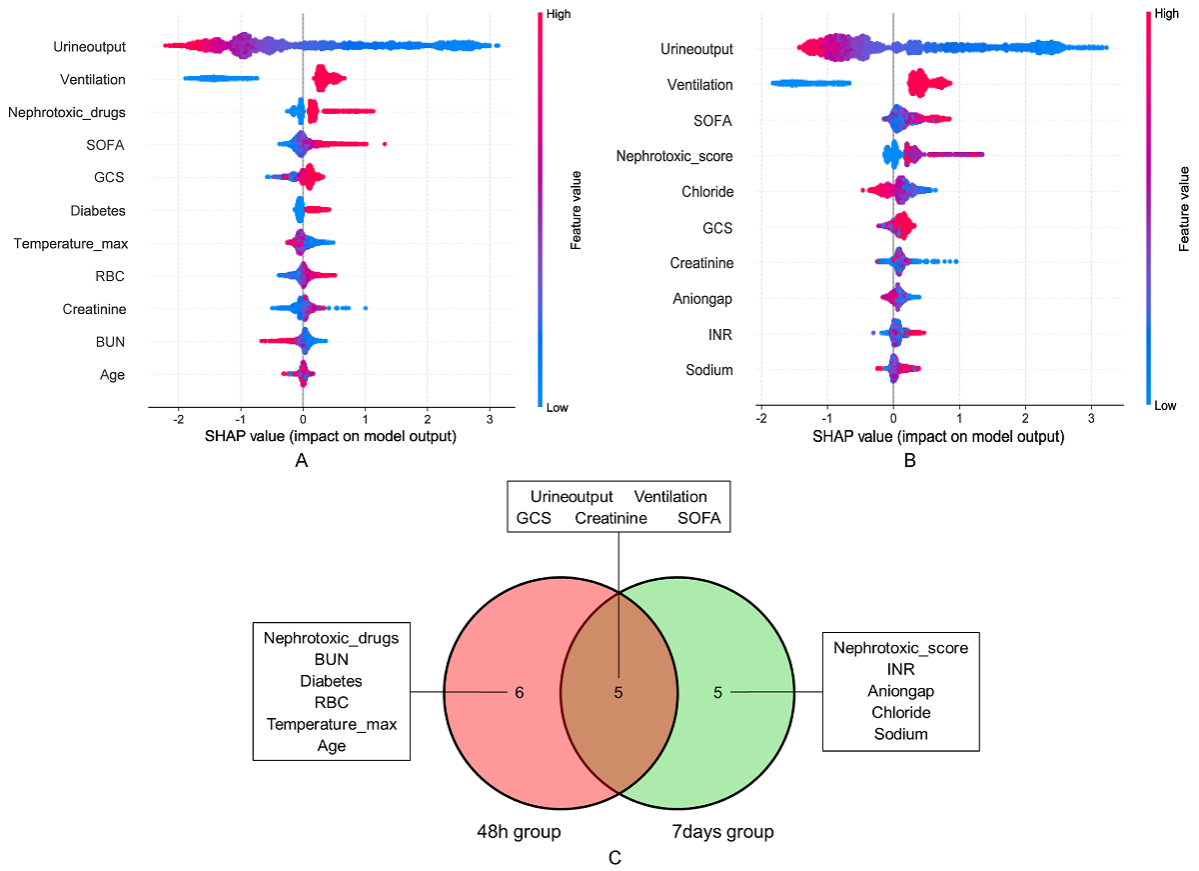
Feature analysis for SA-AKI risk prediction models: (A) 48-hour SHAP summary plot, (B) 7-day SHAP summary plot, and (C) Venn diagram of feature analysis comparing 48-hour and 7-day models. BUN: blood urea nitrogen; GCS: Glasgow Coma Scale; INR: international normalized ratio; Nephrotoxic_drugs: binary indicator of nephrotoxic drug use; Nephrotoxic_score: cumulative score of concurrent nephrotoxic medications (1 point per drug); RBC: red blood cell; SA-AKI: sepsis-associated acute kidney injury; SHAP: Shapley additive explanation; SOFA: Sequential Organ Failure Assessment.

Figure 7A shows that urine output was the most important predictive feature in the 48-hour group, with low urine output (negative SHAP values, blue region) significantly associated with increased AKI risk. Mechanical ventilation, nephrotoxic drug use, SOFA score, Glasgow Coma Scale (GCS) score, and diabetes also demonstrated high importance and were positively correlated with AKI risk. Additionally, maximum temperature, red blood cell (RBC) count, creatinine, BUN, and age provided predictive value in the short term. Figure 7B indicates that in the 7-day group, urine output remained the most important feature, displaying a significant negative correlation consistent with the 48-hour group. Mechanical ventilation and SOFA score continued to serve as important features, indicating the sustained influence of disease severity during the subacute phase. Unlike the 48-hour group, the nephrotoxic drug score replaced individual drug use in the 7-day group, further emphasizing the impact of cumulative drug effects. Additionally, the importance of chloride, anion gap, and sodium significantly increased in the 7-day group, suggesting that electrolyte imbalances and acid-base disturbances may play important roles in the occurrence and progression of kidney injury. SHAP waterfall plots in Multimedia Appendix 13 further demonstrate individualized feature contributions for single patients, intuitively presenting the positive and negative effects of each variable on risk prediction for specific cases.

Figure 7C provides a cross-analysis of features between the 48-hour and 7-day groups, identifying shared and unique characteristics of the two time groups. Urine output, mechanical ventilation, SOFA score, GCS score, and creatinine demonstrated high importance in both groups, representing core shared features for acute and subacute SA-AKI prediction. Additionally, nephrotoxic drug-related features (use and score) are present in different forms during acute and subacute phases, suggesting differential expression across different time dimensions. Furthermore, diabetes, BUN, RBC count, maximum temperature, and age in the 48-hour group were identified as unique features for acute AKI risk, whereas electrolyte indicators (chloride, anion gap, sodium) and INR in the 7-day group contributed more significantly to subacute AKI risk.

**Figure 8:**
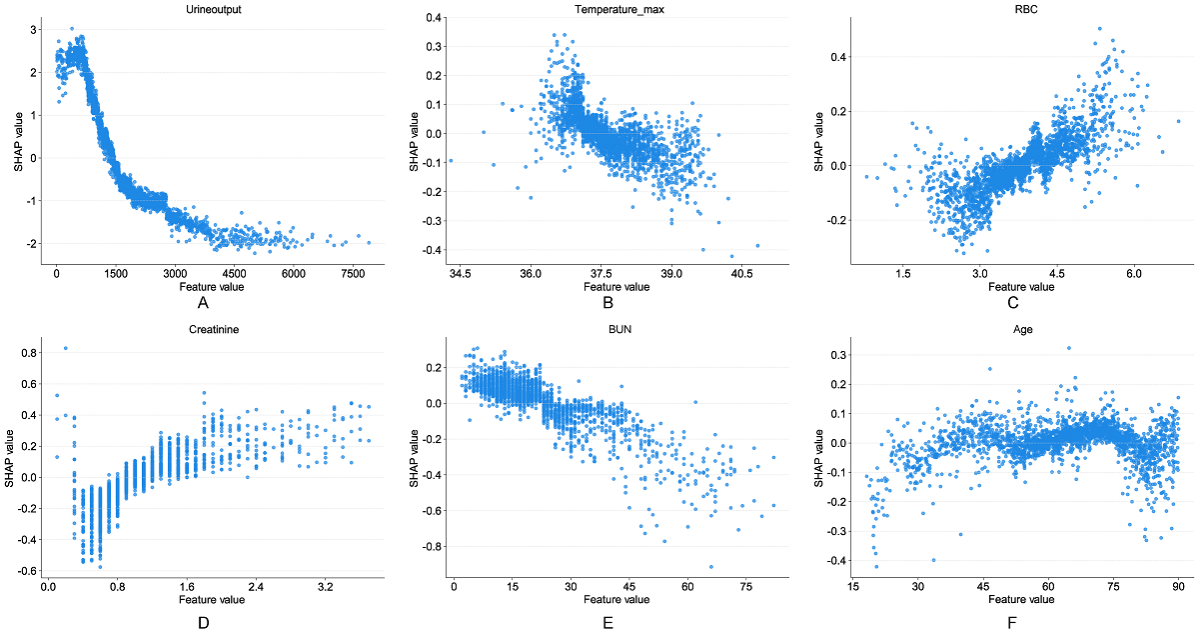
Partial dependence plots of continuous variables in the 48-hour SA-AKI risk prediction model for (A) urine output, (B) maximum temperature, (C) RBC, (D) creatinine, (E) BUN, and (F) Age. BUN: blood urea nitrogen; RBC: red blood cell; SA-AKI: sepsis-associated acute kidney injury; SHAP: Shapley additive explanation.

**Figure 9:**
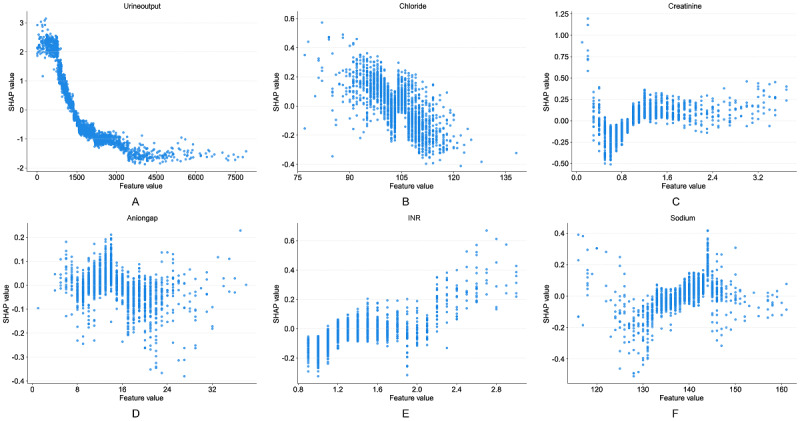
Partial dependence plots of continuous variables in the 7-day SA-AKI risk prediction model for (A) urine output, (B) chloride, (C) creatinine, (D) anion gap, (E) INR, and (F) sodium. INR: international normalized ratio; SA-AKI: Sepsis-associated acute kidney injury; SHAP: Shapley additive explanation.

Figures 8 and 9, respectively, demonstrate the nonlinear relationships between continuous variables and SA-AKI risk in the 48-hour and 7-day prediction groups, with complete feature dependence plots for all model variables detailed in Multimedia Appendices 14 and 15. In the 48-hour prediction model, urine output (Figure 8A) was the most important predictor, showing a nonlinear negative correlation with SA-AKI risk. When 24-hour urine output fell below 1500 mL, risk began to increase, with SHAP values rising sharply above 3.0 when below 1000 mL. Maximum temperature (Figure 8B) showed positive SHAP values in the 36.0-37.5 °C range, with SHAP values turning negative and gradually declining after exceeding 38 °C. RBC count (Figure 8C) demonstrated a positive correlation with SA-AKI risk, with SHAP values turning positive and gradually increasing when RBC levels exceeded 4.5×10^12^/L. SCr (Figure 8D) exhibited a clear threshold effect, with the risk inflection point occurring at approximately 0.8 mg/dL, after which SHAP values continued to rise. Additionally, BUN (Figure 8E) showed a unique negative correlation pattern with an inflection point at approximately 20 mg/dL, while age (Figure 8F) displayed an increasing risk trend in older patients. In the 7-day prediction model, urine output (Figure 9A) remained the most critical negatively correlated variable, with the threshold adjusted upward to approximately 1500 mL and showing a steeper risk gradient in the low urine output region. Chloride (Figure 9B) exhibited a typical inverted U-shaped relationship, with SHAP values approaching zero in the normal range of 100-110 mmol/L and significant risk changes when deviating from this range. SCr (Figure 9C) threshold was near 0.8 mg/dL, essentially consistent with the 48-hour model. Anion gap (Figure 9D; threshold 20 mmol/L) and INR (Figure 9E; threshold 1.5) were both positively correlated with risk; blood sodium concentration (Figure 9F) remained stable within the normal physiological range, with hypernatremia increasing SA-AKI risk.

### Model Application

**Figure 10:**
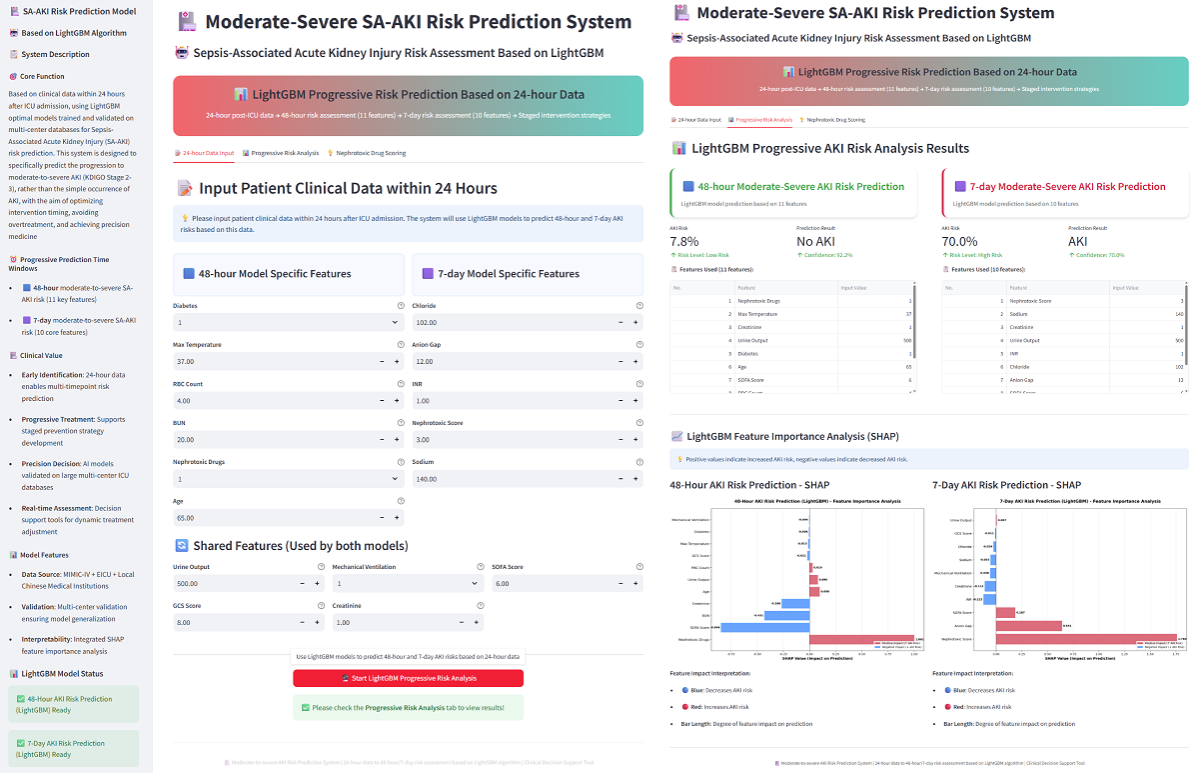
User interface and example output of the web-based SA-AKI risk prediction app. AKI: acute kidney injury; ICU: intensive care unit; KDIGO: Kidney Disease: Improving Global Outcomes; LightGBM: light gradient boosting machine; MIMIC-IV: Medical Information Mart for Intensive Care-IV; SA-AKI: sepsis-associated acute kidney injury; SHAP: Shapley additive explanation.

To translate our prediction models into clinical decision support tools usable in practice, we deployed the optimal 48-hour and 7-day LightGBM models as a publicly accessible web-based app [26]. This tool, developed using the Streamlit framework, features a clean and intuitive user interface that allows clinicians to quickly input key patient variables. After data submission, the system not only instantly calculates and displays SA-AKI risk probabilities for both 48-hour and 7-day time points, but its core advantage lies in simultaneously generating individualized SHAP force plots (Figure 10). This design presents prediction results alongside their underlying drivers in an intuitive side-by-side format, addressing traditional model “black box” limitations and aiming to provide transparent, interpretable decision support for precise clinical interventions.

## Discussion

### Principal Findings

This study systematically analyzed the dynamic progression of SA-AKI, identifying 48 hours and 7 days as critical time points representing distinct phases of disease evolution: rapid deterioration during the acute phase and cumulative disease progression effects in the subacute phase. Based on these time points, we developed and validated a stratified, dynamic prediction system using multisource databases. We compared the model with 8 machine learning algorithms, including CatBoost and TabNet, ultimately selecting the LightGBM model, which demonstrated consistent performance across discrimination metrics, generalizability testing, and clinical net benefit analysis. The model’s accuracy was confirmed in the MIMIC-IV training set (48-h/7-d AUCs of 0.862/0.922, respectively), and its generalizability was validated across three heterogeneous validation cohorts: first, satisfactory performance on the MIMIC-IV internal test set (AUCs of 0.839/0.834, respectively); second, consistent performance in external validation of the North American multicenter eICU-CRD cohort (AUCs: 0.770/0.720), and Chinese single-center FAH-HMU cohort (AUCs: 0.793/0.773). Furthermore, the model’s robustness and fairness were confirmed through systematic subgroup analyses, demonstrating consistent discriminative ability across different age, gender, and comorbidity subgroups. To achieve clinical translation of the research findings, we combined SHAP analysis to enhance model transparency and deployed it as a publicly accessible web-based app, enabling clinicians to assess high-risk patients and develop individualized intervention strategies. This study addresses the existing gap in stratified prediction for moderate-to-severe AKI and provides a management decision support system that has undergone multiregional, multicenter validation and combines interpretability with clinical usability.

### Comparison to Prior Work

Previous studies [20,21,27,28] have largely focused on overall AKI risk prediction, with insufficient attention to early, precise prediction of moderate-to-severe AKI (stages 2/3), potentially leading to overtreatment of mild cases or interruption of effective treatment regimens. The 15th Acute Kidney Injury Quality Initiative consensus [29] clearly states that early prediction of moderate-to-severe AKI can more effectively guide clinical treatment decisions and improve patient outcomes. Takkavatakarn et al [30] analyzed SCr trajectories in patients with sepsis using the MIMIC-IV database and found that approximately 43.68% of patients with sepsis had mild renal dysfunction that could recover in the short term, indicating that existing nonstratified prediction models may pose risks of overtreatment.

Although the KDIGO guidelines include both SCr and urine output as joint diagnostic indicators for AKI, most current machine learning prediction models are based solely on SCr and do not fully incorporate the dynamic changes in urine output, which may lead to bias and risk omission in final prediction results. For example, Martinez et al [31] showed that an SCr-based machine learning model achieved an AUC of 0.81 (95% CI 0.80-0.82) in predicting AKI progression, but due to the lag and volatility of SCr, it was difficult to sensitively capture early pathological changes. Similarly, another study of patients after cardiac surgery [32] indicated that a model solely dependent on SCr, despite achieving an AUC of 0.843 (95% CI 0.778-0.899), may miss some high-risk patients due to the lack of integration of indicators such as urine output. Urine output, as another key indicator, can sensitively reflect early renal function impairment through its dynamic changes. Macedo et al [33] found that urine output–based diagnostic criteria had higher sensitivity compared to SCr and could identify more AKI cases, but their stability was slightly inferior to SCr; Martins et al [34] further indicated that combining urine output with SCr could significantly improve diagnostic rates (18% improvement). Based on these research findings, this study’s diagnostic criteria innovatively combine urine output and SCr, two core indicators, compensating for the limitations of single indicators and significantly enhancing the model’s sensitivity and comprehensiveness in identifying patients with AKI.

### Interpretation of Key Predictive Features

Through cross-validation analysis (Figure 7C), this study identified urine output, SOFA score, mechanical ventilation, creatinine, GCS score, and nephrotoxic drug use as common core features in both the 48-hour and 7-day prediction models, further validating the universality of these features in predicting SA-AKI risk across different stages. Notably, the dynamic changes in urine output and SOFA score consistently ranked among the top four features in importance ranking at both time points (Figures 7A and 7B). When urine output significantly decreased (48-h group threshold <1000 mL, 7-d group threshold <1500 mL), it was closely associated with increased AKI risk (Figures 8A and 9A); whereas the SOFA score comprehensively revealed the cumulative effects of multiorgan dysfunction and systemic inflammatory responses [35,36].

Mechanical ventilation emerged as another key feature, suggesting that hypoxemia or ventilation-induced lung injury may exacerbate renal tubular ischemia-reperfusion injury and further impair kidney function [37]. In comparison, the influence of nephrotoxic drugs showed different characteristics across different time ranges. In the 48-hour prediction model, whether nephrotoxic drugs were used was a key variable, reflecting their direct short-term effects on renal function; whereas in the 7-day prediction model, the number of combined drug uses was more important, emphasizing that the cumulative effects of drugs play a more critical role in long-term renal function deterioration.

Furthermore, time-specific features further revealed the regularity of stage-dependent disease progression. The 48-hour prediction model focused on acute pathological changes, with key features including BUN, RBC count, and maximum body temperature. Notably, BUN showed a negative correlation with AKI risk (Figure 8E), which contradicts traditional expectations and may be related to factors such as fluid overload, hypoproteinemia, and hepatic compensation. Meanwhile, increased RBC count was positively correlated with SA-AKI risk (Figure 8C), potentially reflecting the negative impact of blood concentration and hemodynamic changes on renal microcirculation under acute stress conditions. The negative correlation between elevated body temperature and SA-AKI risk (Figure 8B) may suggest that hyperthermia reflects the body’s remaining immune response capacity and metabolic reserves, while the absence of fever may indicate more severe circulatory failure. These features collectively revealed the characteristics of rapid deterioration in the acute phase. In contrast, the 7-day prediction model focused more on subacute-phase metabolic disturbances and cumulative pathological changes, with significant features including anion gap, chloride, sodium, and INR (Figures 9B and 9D-9F). Among these, abnormalities in anion gap, chloride, and sodium suggest the critical role of electrolyte imbalances and acid-base disturbances in renal injury progression, whereas elevated INR reflects the possibility of coagulopathy and microcirculatory dysfunction [38].

### Subgroup Analysis

The subgroup analysis of this study confirmed that our models demonstrated broad robustness and fairness in both internal (MIMIC-IV) and external (eICU-CRD) validation (Figure 6A,B). The models showed consistent high discriminative ability across key subgroups defined by age, gender, and major comorbidities, without significant statistical heterogeneity. This provides strong evidence in support of model application in diverse ICU patient populations. However, the “anomalous” high AUC values observed in the SLD subgroup during external validation, accompanied by extremely high heterogeneity (*I*²>74%), warrant careful consideration. We believe this is very likely not a true reflection of superior model performance, but rather a statistical artifact caused by the extremely small sample size of this subgroup. Since patients with SLD constitute only a very small proportion of the total population (1%-2%), their unique pathophysiological processes may lead to a distribution of predicted probabilities that differs significantly from the overall cohort, thereby causing AUC value distortion and overestimation under small sample conditions. Therefore, current models should be applied with particular caution to patients with SLD. Future work needs targeted validation in large-scale cohorts of patients with cirrhosis or liver failure to clarify their true utility in this special population.

### Strengths

This study constructed a “dual time-point” prediction model in which 48 hours and 7 days are used to represent critical nodes of the acute and subacute phases, respectively. This model not only captures the distinctive characteristics of disease progression at different stages but also highlights the intervention value of time points. The core of the 48-hour prediction model lies in “rapid identification and timely intervention,” providing a scientific basis for rapid disease deterioration, whereas the 7-day model emphasizes “disease monitoring and strategy adjustment,” which focuses on the management of long-term cumulative effects of disease progression. Compared to previous single time-point research models, this study comprehensively analyzed the differences and commonalities between acute and subacute phase characteristics, addressing the deficiencies in temporal dimension research and providing clinicians with a medical management tool covering acute to subacute phases. Additionally, this model demonstrated robust performance across three heterogeneous cohorts: North American multicenter (eICU-CRD), American single-center (MIMIC-IV), and Chinese single-center (FAH-HMU), and through subgroup analysis further confirmed its generalizability across different populations and regions. Finally, we translated our research findings into a publicly accessible, web-based app integrated with real-time SHAP analysis, facilitating translation from research to clinical application.

### Limitations

Although this study has made important progress in SA-AKI stage-specific prediction, several limitations remain that warrant further exploration. First, this study was based on retrospective data for model development and validation. Although this study analyzed patients with ICU stays >48 hours and complete AKI diagnostic data within 28 days to ensure data integrity as much as possible, the study did not include patients who left the ICU early due to rapid clinical deterioration or death, potentially leading to the omission of some disease progression patterns. Second, the study samples mainly came from MIMIC-IV and eICU-CRD databases, and their applicability to other regional populations needs careful evaluation. Although the model performed robustly in the Chinese single-center cohort (FAH-HMU), this cohort had a relatively small sample size and may have been subject to bias. Therefore, a large-scale, multicenter prospective clinical trial remains the gold standard for ultimately confirming the model’s clinical applicability. Additionally, this study’s dynamic features had relatively low temporal resolution, selecting only two key time points—48 hours and 7 days—and could not fully explore the predictive value of additional time points. Finally, although the model integrated core features such as urine output, mechanical ventilation, nephrotoxic drugs, and SOFA score, feature selection was still limited to routine electronic medical record data and lacked integration of higher-dimensional biomarkers (such as NGAL and KIM-1) and real-time clinical data.

### Future Directions

Future research should validate the model’s clinical applicability through prospective multicenter studies to further enhance its generalizability. Additionally, multitime-point dynamic data integration analysis could be introduced, comprehensively capturing dynamic disease progression through continuous monitoring of patients’ pathophysiological indicators, further improving the model’s predictive performance and clinical utility. Furthermore, as this study was mainly based on static data, future research could introduce multitime-point dynamic data and other multidimensional information, even integrating the model with local hospital electronic health record systems through continuous learning for real-time training and calibration, making the model more aligned with regional epidemiological characteristics and medical practice needs, thereby enhancing the model’s precision and applicability.

### Conclusions

This study developed and validated a dynamic, stratified, dual-time-point prediction system for predicting moderate-to-severe SA-AKI based on multisource databases. The system, centered on LightGBM, demonstrated performance, robustness, and generalizability through benchmark comparisons with advanced algorithms such as CatBoost and TabNet, as well as validation in multicenter and multiregional cohorts in North America and China. This study provides a stratified, interpretable, and stage-specific decision support framework that differs from traditional single risk scoring approaches by covering both acute and subacute phases for SA-AKI clinical management. Web-based app may provide further risk stratification in clinical applications.

## Appendix

Multimedia Appendix 1 Flowchart for the selection of sepsis patients with SA-AKI from FAH-HMU ICU database for external validation. AKI: acute kidney injury; ICU: intensive care unit; RRT: renal replacement therapy; SA-AKI: sepsis-associated acute kidney injury; FAH-HMU: First Affiliated Hospital of Hainan Medical University.

Multimedia Appendix 2 Workflow of machine learning model construction and validation for predicting SA-AKI using MIMIC-IV training, eICU-CRD and FAH-HMU ICU external validation datasets. SA-AKI: Sepsis-associated acute kidney injury; SMOTE: Synthetic Minority Oversampling Technique; LightGBM: Light Gradient Boosting Machine; SHAP: Shapley additive explanation.

Multimedia Appendix 3 Staging criteria for sepsis-associated acute kidney injury based on KDIGO guidelines with serum creatinine and urine output thresholds for stages 0-3. KDIGO: Kidney Disease: Improving Global Outcomes; RRT: renal replacement therapy; SA-AKI: sepsis-associated acute kidney injury.

Multimedia Appendix 4 Distribution of nephrotoxic medications and number of users in sepsis patients with SA-AKI from MIMIC-IV database.

Multimedia Appendix 5 Heatmap of SA-AKI stage progression patterns from 24 hours to 48 hour and 7 day in MIMIC-IV database. (A) Progression patterns for patients with initial AKI stage 0 at 24 hours; (B) Progression patterns for patients with initial AKI stage 1 at 24 hours; (C) Progression patterns for patients with initial AKI stage 2 at 24 hours; (D) Progression patterns for patients with initial AKI stage 3 at 24 hours.

Multimedia Appendix 6 Heatmap of SA-AKI stage progression patterns from 24 hours to 48 hour and 7 day in eICU-CRD database. (A) Progression patterns for patients with initial AKI stage 0 at 24 hours; (B) Progression patterns for patients with initial AKI stage 1 at 24 hours; (C) Progression patterns for patients with initial AKI stage 2 at 24 hours; (D) Progression patterns for patients with initial AKI stage 3 at 24 hours.

Multimedia Appendix 7 Complete baseline characteristics of patients with SA-AKIa by progression to moderate-to-severe SA-AKI (stage 2-3) within 48 hours and 7 days (MIMIC-IV database).

Multimedia Appendix 8 Feature importance comparison between 48-hour and 7-day SA-AKI prediction models using SHAP values and LightGBM rankings. (A) SHAP importance ranking for 48-hour model; (B) SHAP value distribution for 48-hour model; (C) LightGBM feature importance for 48-hour model; (D) SHAP importance ranking for 7-day model; (E) SHAP value distribution for 7-day model; (F) LightGBM feature importance for 7-day model. SA-AKI: sepsis-associated acute kidney injury; SHAP: Shapley additive explanation; LightGBM: Light Gradient Boosting Machine.

Multimedia Appendix 9 Decision curve analysis and calibration plots for SA-AKI risk prediction models in FAH-HMU ICU external validation cohort. (A) Decision curve analysis for 48-hour model; (B) Calibration plot for 48-hour model; (C) Decision curve analysis for 7-day model; (D) Calibration plot for 7-day model. DCA: Decision curve analysis; RF: Random forest; XGBoost: Extreme Gradient Boosting; CatBoost: Categorical Boosting; LightGBM: Light Gradient Boosting Machine; SA-AKI: Sepsis-associated acute kidney injury.

Multimedia Appendix 10 Performance comparison of different model frameworks for dual-timepoint SA-AKI prediction using DeLong test in MIMIC-IV internal validation cohort.

Multimedia Appendix 11 Subgroup analysis of 48-hour SA-AKI risk prediction model performance in MIMIC-IV internal validation cohort. AUC: Area under the curve; CI: Confidence interval; CHF: Congestive heart failure; SLD: Severe liver disease; SA-AKI: Sepsis-associated acute kidney injury.

Multimedia Appendix 12 Subgroup analysis of 7-day SA-AKI risk prediction model performance in MIMIC-IV internal validation cohort. AUC: Area under the curve; CI: Confidence interval; CHF: Congestive heart failure; SLD: Severe liver disease; SA-AKI: Sepsis-associated acute kidney injury.

Multimedia Appendix 13 SHAP waterfall plots demonstrating individual patient-level feature contributions for SA-AKI risk prediction using LightGBM models. (A) 48-hour model waterfall plot for high-risk prediction (towards Class 1); (B) 48-hour model waterfall plot for low-risk prediction (towards Class 0); (C) 7-day model waterfall plot for high-risk prediction (towards Class 1); (D) 7-day model waterfall plot for low-risk prediction (towards Class 0). SA-AKI: Sepsis-associated acute kidney injury; SHAP: Shapley additive explanation; LightGBM: Light Gradient Boosting Machine; SOFA: Sequential Organ Failure Assessment; GCS: Glasgow Coma Scale; BUN: Blood urea nitrogen; INR: International normalized ratio.

Multimedia Appendix 14 Complete SHAP dependence plots for all features in 48-hour SA-AKI risk prediction model showing individual feature-outcome relationships. (A) Urine output; (B) Ventilation; (C) Nephrotoxic drugs; (D) SOFA score; (E) GCS score; (F) Diabetes; (G) Maximum temperature; (H) RBC count; (I) Creatinine; (J) BUN; (K) Age.

Multimedia Appendix 15 Complete SHAP dependence plots for all features in 7-day SA-AKI risk prediction model showing individual feature-outcome relationships. (A) Urine output; (B) Ventilation; (C) SOFA score; (D) Nephrotoxic score; (E) Chloride; (F) GCS score; (G) Creatinine; (H) Anion gap; (I) INR; (J) Sodium.

## Abbreviations

AKI: acute kidney injury
AUC: area under the receiver operating characteristic curve
BUN: blood urea nitrogen
DCA: decision curve analysis
eICU-CRD: electronic intensive care unit collaborative research database
FAH-HMU: First Affiliated Hospital of Hainan Medical University
GCS: Glasgow Coma Scale
ICU: intensive care unit
INR: international normalized ratio
KDIGO: Kidney Disease: Improving Global Outcomes
LightGBM: light gradient boosting machine
MIMIC-IV: Medical Information Mart for Intensive Care, version IV
RBC: red blood cell
SA-AKI: sepsis-associated acute kidney injury
SCr: serum creatinine
SHAP: Shapley Additive explanations
SLD: severe liver disease
SOFA: Sequential Organ Failure Assessment
XGBoost: extreme gradient boosting

## Data Availability

The datasets generated or analyzed during this study have mixed availability. Two publicly available datasets were used: MIMIC-IV is available in the PhysioNet repository [24], and the eICU Collaborative Research Database is available in the MIT Laboratory for Computational Physiology repository [25]. The dataset from the First Affiliated Hospital of Hainan Medical University ICU is not publicly available due to patient privacy and institutional data-sharing restrictions but may be available from the corresponding author on reasonable request with appropriate ethical approval and data use agreements. The web-based prediction app developed in this study is publicly accessible [26]. All analysis code and model implementation details are available from the corresponding author on reasonable request.
