# Supplementary figures and images for: Prediction of Moderate-to-Severe Sepsis-Associated Acute Kidney Injury Using a Dual-Timepoint Machine Learning Model: Development, Multiregional Validation, and Clinical Deployment Study
Source: J Med Internet Res. 2025 Sep 30;27:e73840. doi: 10.2196/73840 (PMC12521856; doi:10.2196/73840)

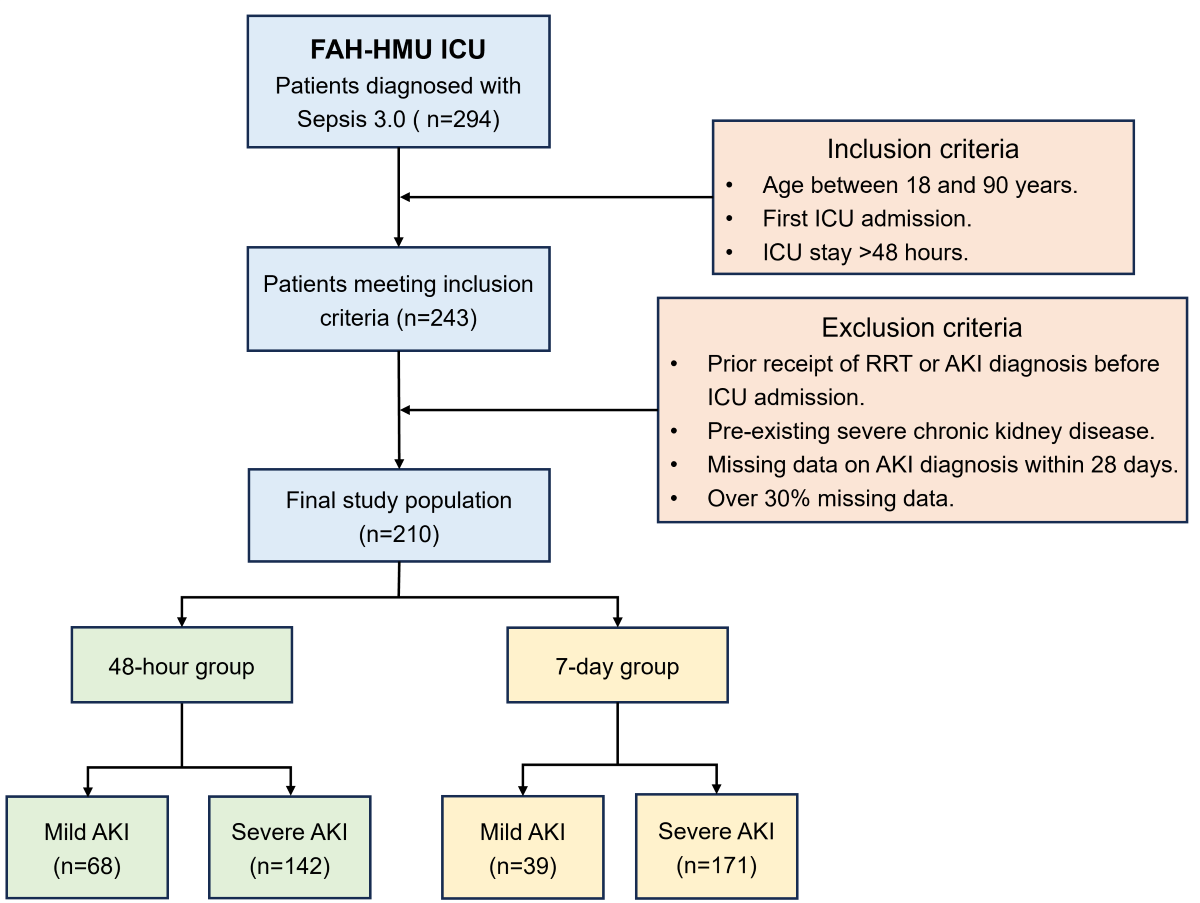

Supplement: Multimedia Appendix 1 [file jmir_v27i1e73840_app1.png]

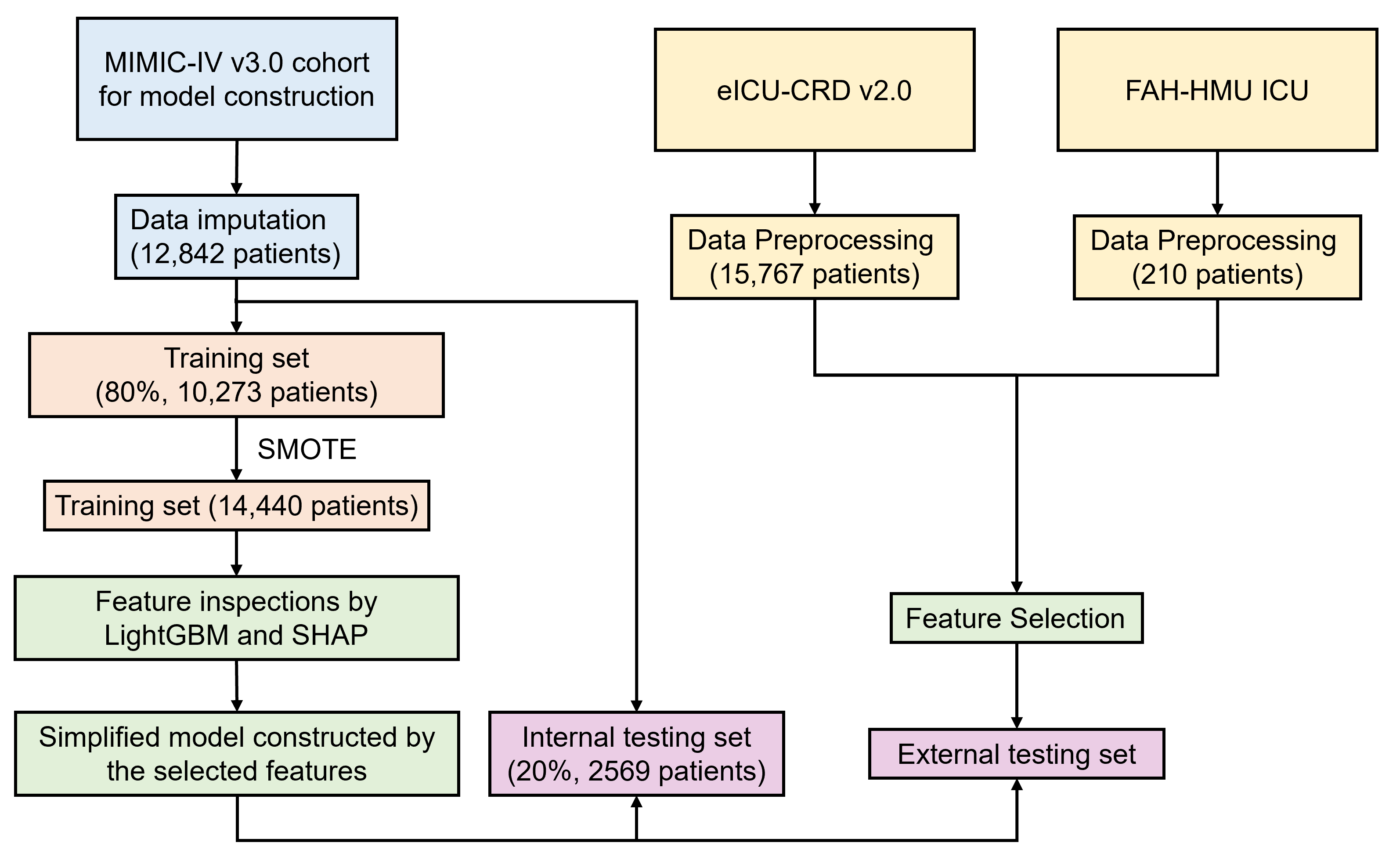

Supplement: Multimedia Appendix 2 [file jmir_v27i1e73840_app2.png]

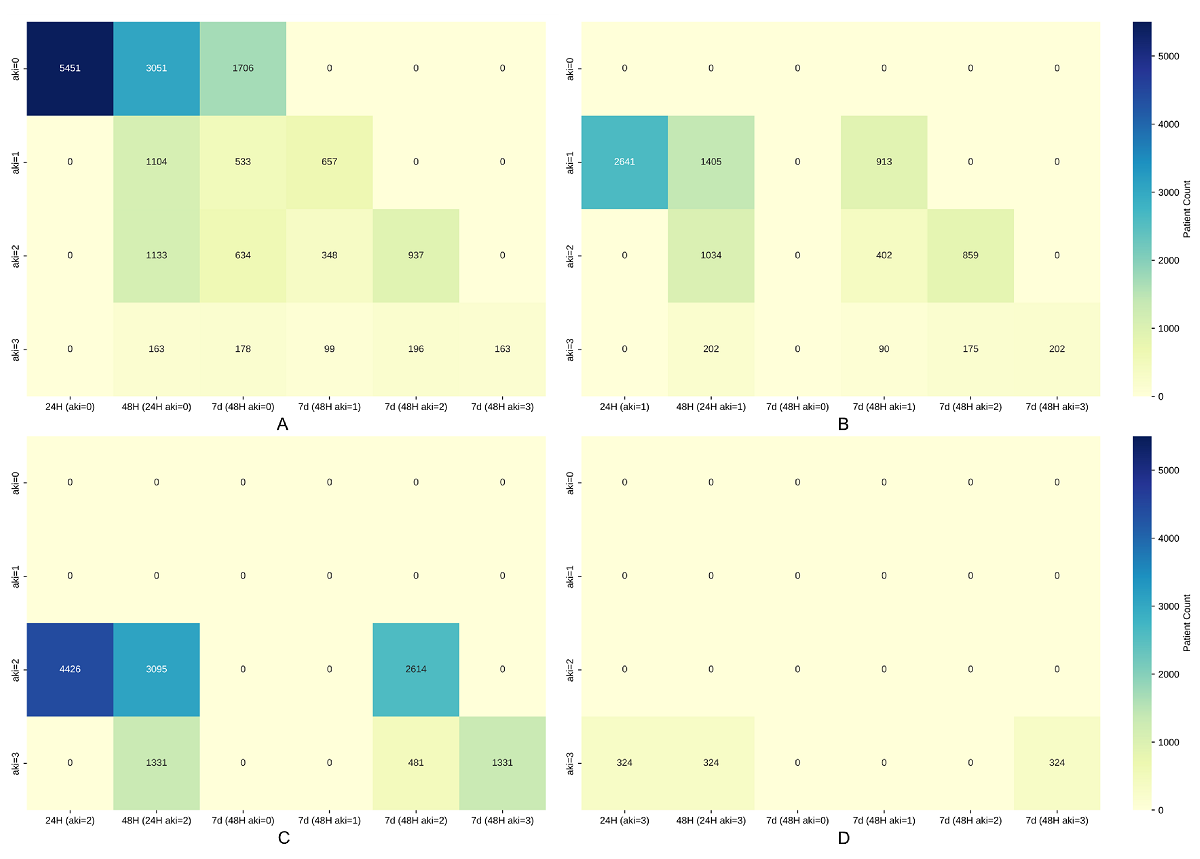

Supplement: Multimedia Appendix 5 [file jmir_v27i1e73840_app5.png]

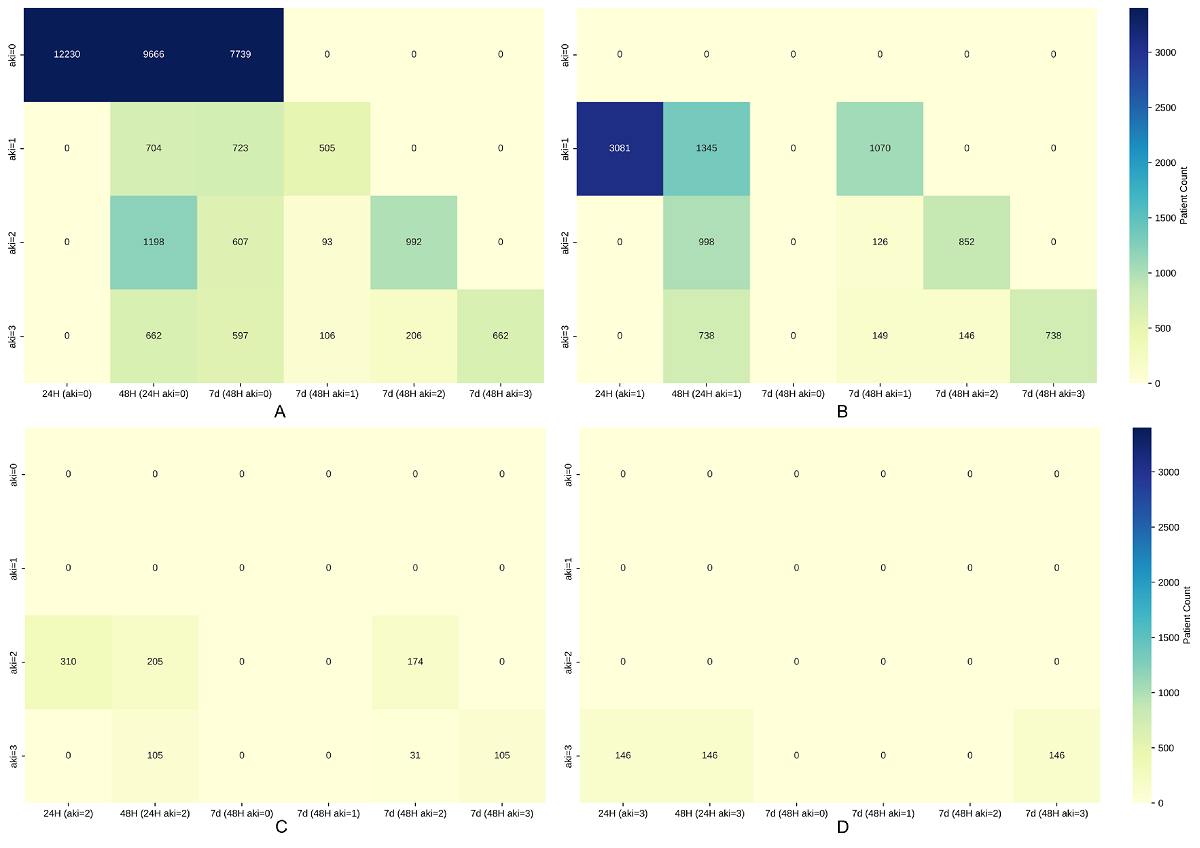

Supplement: Multimedia Appendix 6 [file jmir_v27i1e73840_app6.png]

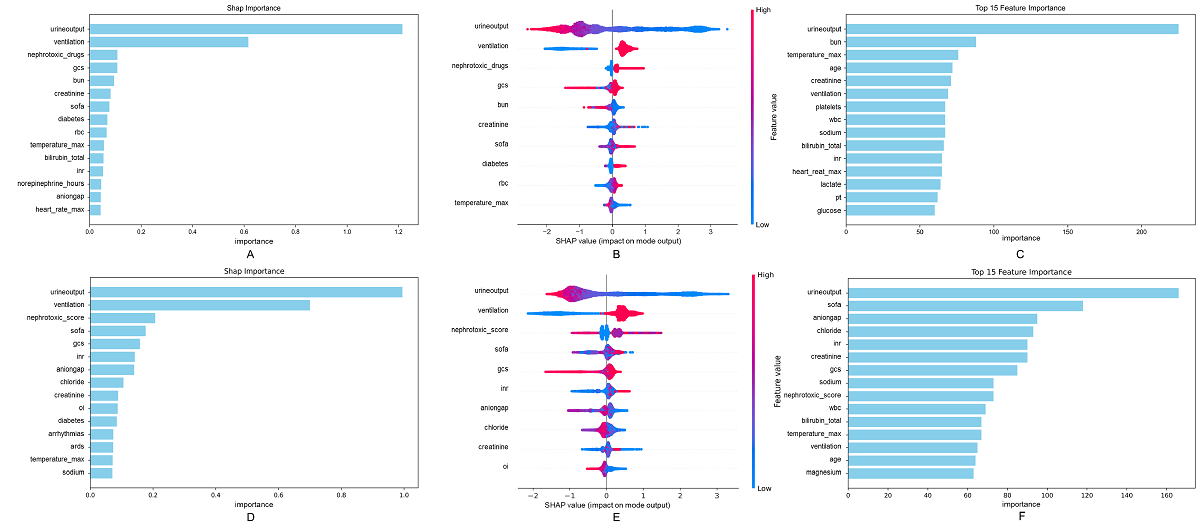

Supplement: Multimedia Appendix 8 [file jmir_v27i1e73840_app8.png]

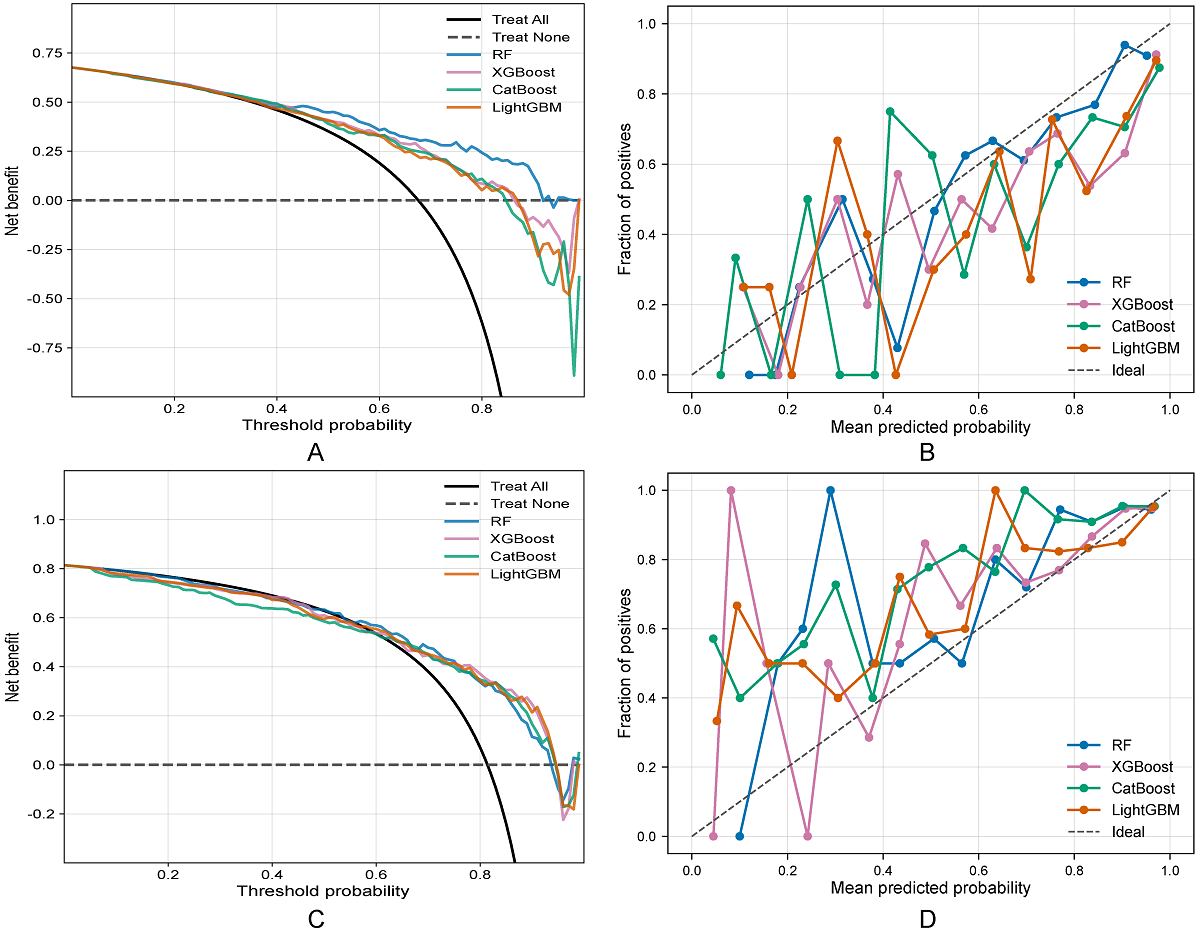

Supplement: Multimedia Appendix 9 [file jmir_v27i1e73840_app9.png]

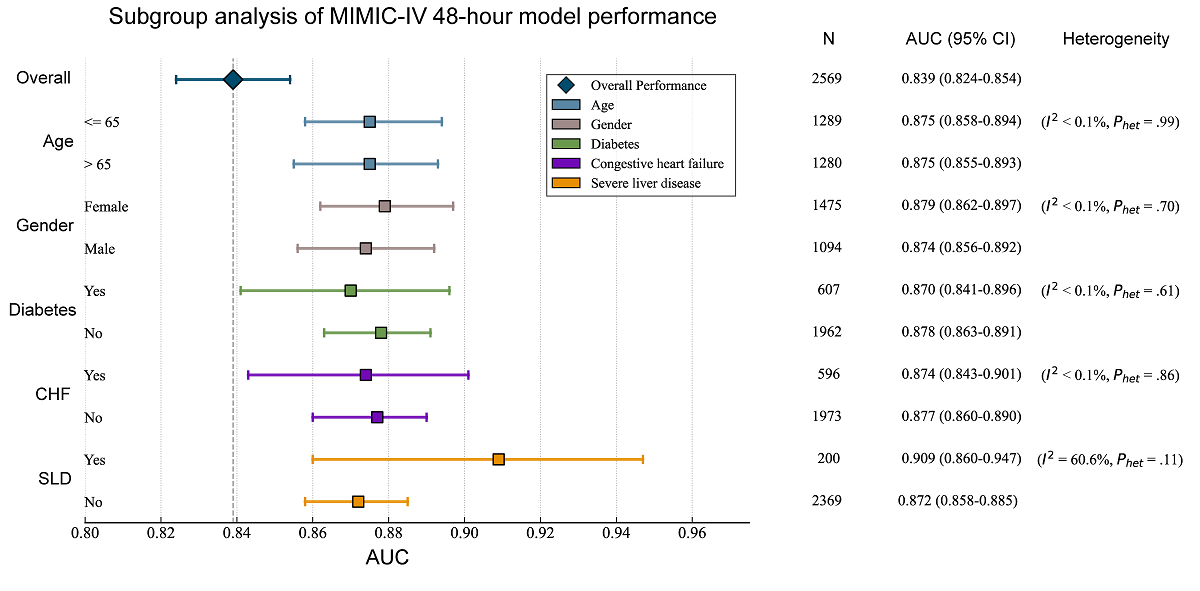

Supplement: Multimedia Appendix 11 [file jmir_v27i1e73840_app11.png]

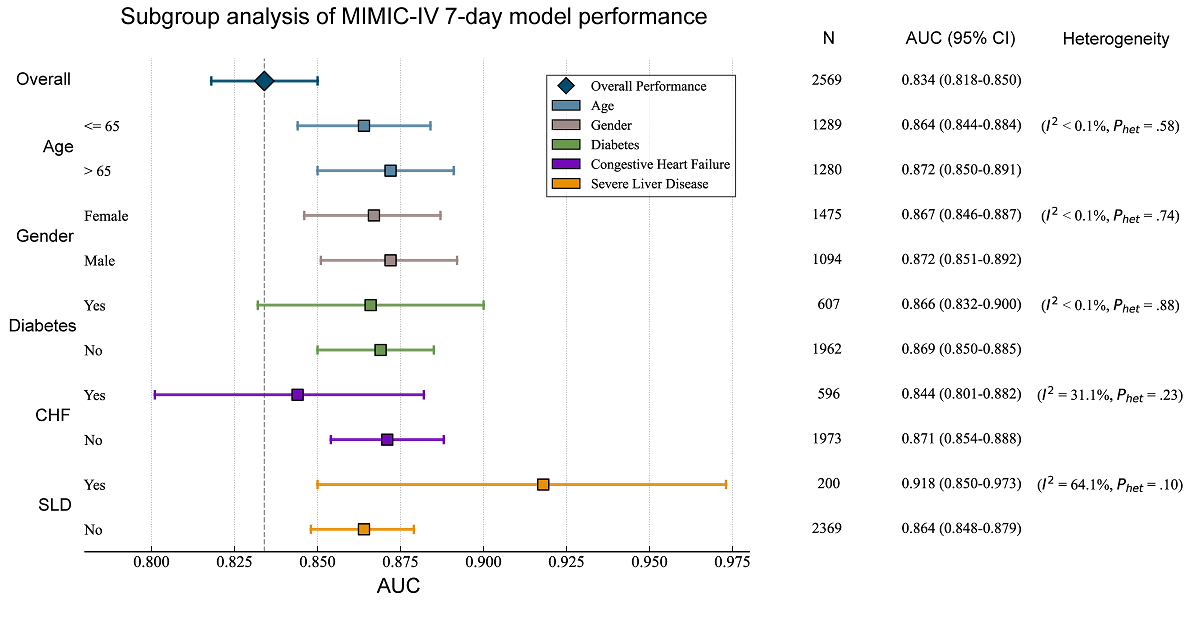

Supplement: Multimedia Appendix 12 [file jmir_v27i1e73840_app12.png]

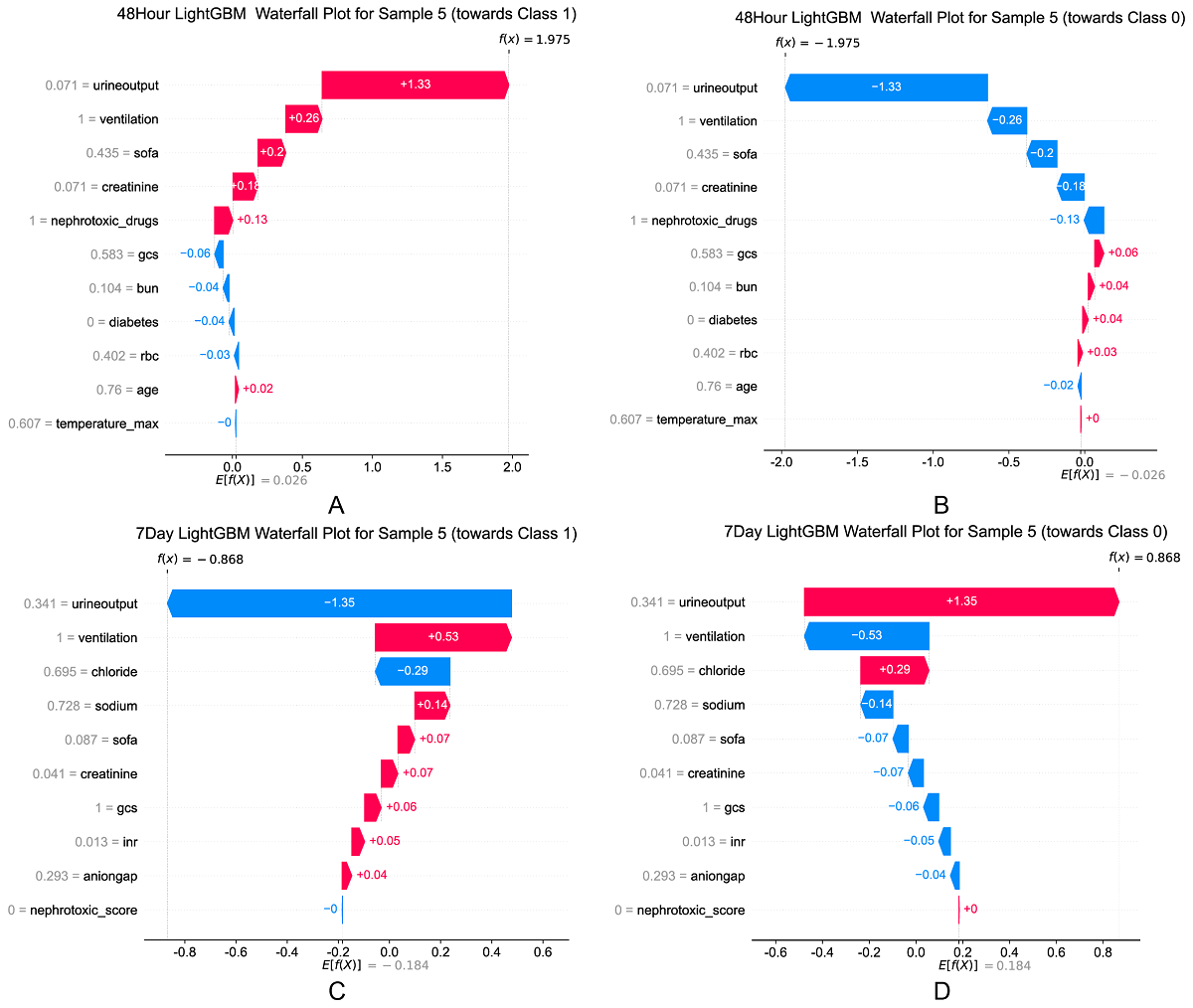

Supplement: Multimedia Appendix 13 [file jmir_v27i1e73840_app13.png]

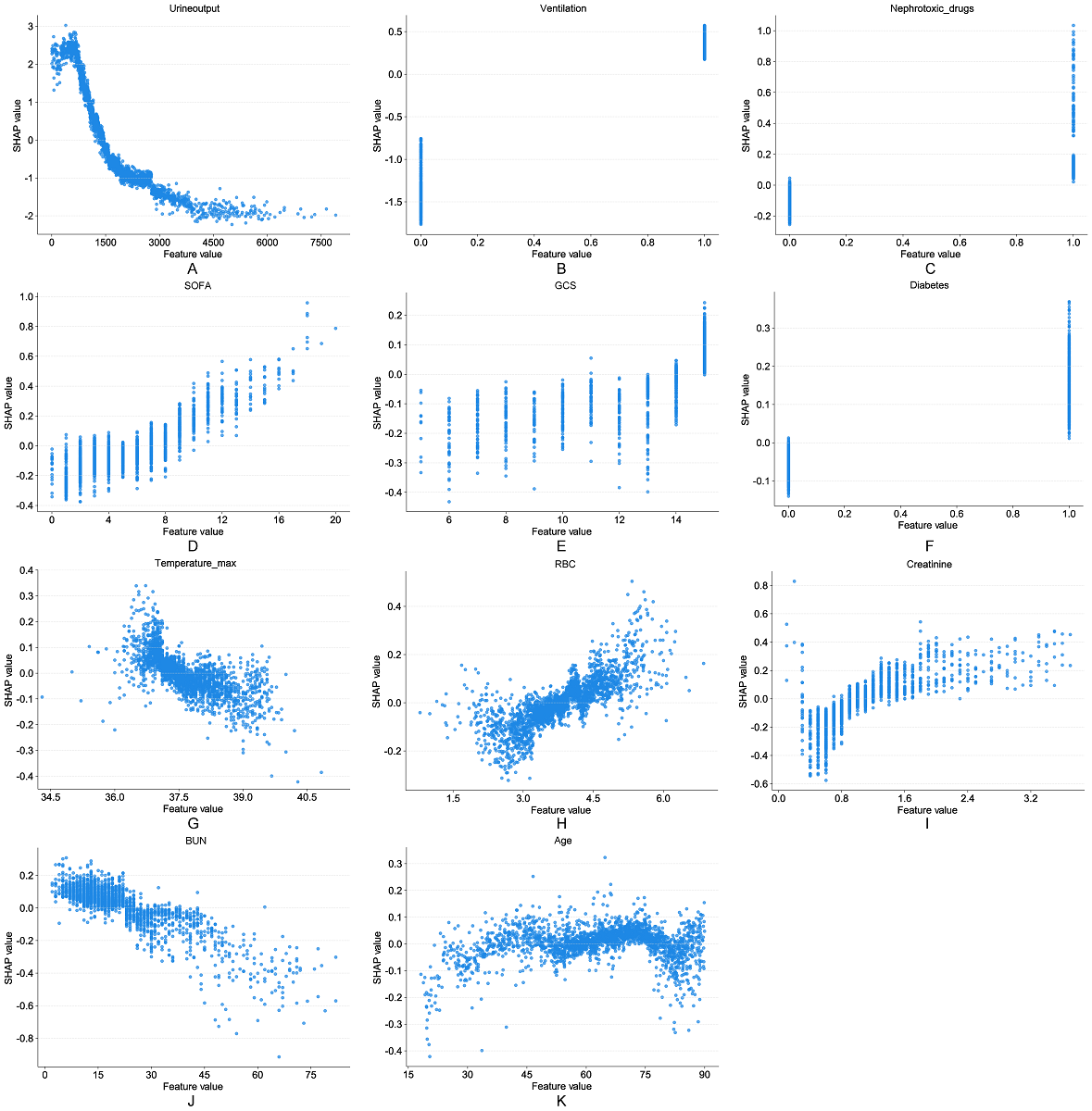

Supplement: Multimedia Appendix 14 [file jmir_v27i1e73840_app14.png]

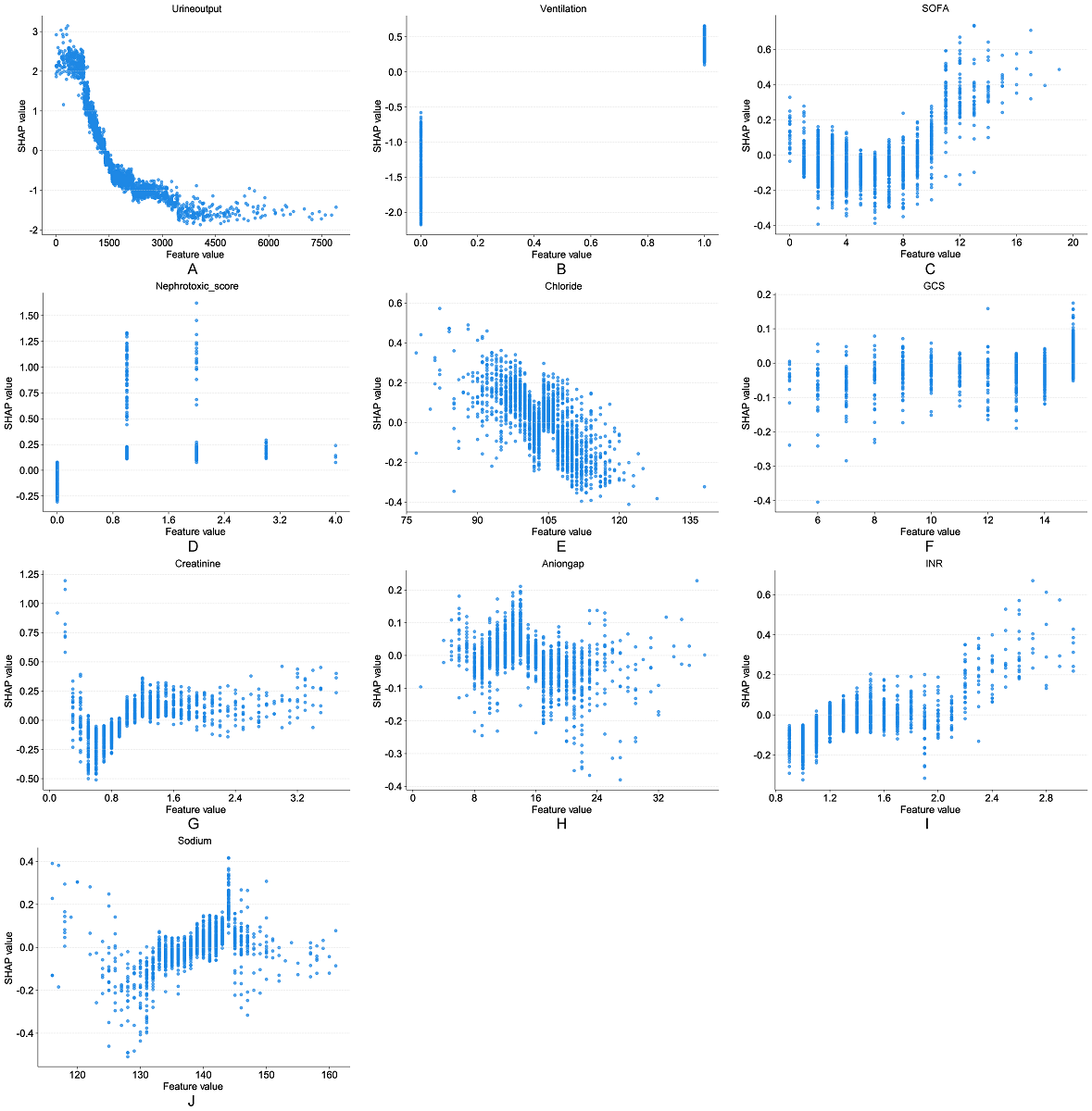

Supplement: Multimedia Appendix 15 [file jmir_v27i1e73840_app15.png]
