# Supplementary material for: Prediction of Moderate-to-Severe Sepsis-Associated Acute Kidney Injury Using a Dual-Timepoint Machine Learning Model: Development, Multiregional Validation, and Clinical Deployment Study
Source: J Med Internet Res. 2025 Sep 30;27:e73840. doi: 10.2196/73840 (PMC12521856; doi:10.2196/73840)
Supplement: Multimedia Appendix 3 [file jmir_v27i1e73840_app3.docx]

| **Stage** | **Definition** | **Serum Creatinine Criteria** | **Urine Output Criteria** |
| --- | --- | --- | --- |
| 0^a^ | Normal kidney function | No change or slight increase | ≥0.5 mL/kg/h for ≥6 hours |
| 1^b^ | Mild injury | 1. Increase ≥0.3 mg/dL within 48 hours  2. 1.5–1.9× baseline within 7 days | <0.5 mL/kg/hr for 6–12 hours |
| 2^b^ | Moderate injury | 2.0–2.9× baseline | <0.5 mL/kg/hr for ≥12 hours |
| 3^b^ | Severe injury | 1. ≥3.0× baseline  2. Increase to ≥4.0 mg/dL  3. Initiation of renal replacement therapy (RRT) | 1. <0.3 mL/kg/hr for ≥24 hours  2. Anuria for ≥12 hours |

^a^ Stage 0: Normal kidney function, not included in the KDIGO guidelines.

^b^ Stages 1–3: Defined according to the 2012 KDIGO guidelines.
