# Supplementary material for: Prediction of Moderate-to-Severe Sepsis-Associated Acute Kidney Injury Using a Dual-Timepoint Machine Learning Model: Development, Multiregional Validation, and Clinical Deployment Study
Source: J Med Internet Res. 2025 Sep 30;27:e73840. doi: 10.2196/73840 (PMC12521856; doi:10.2196/73840)
Supplement: Multimedia Appendix 4 [file jmir_v27i1e73840_app4.docx]

| **Medication Name** | **Number of Users (n)** |
| --- | --- |
| Vancomycin | 5175 |
| Aspirin | 3043 |
| Acyclovir | 515 |
| Neomycin | 210 |
| Gentamicin | 142 |
| Tacrolimus | 74 |
| Ibuprofen | 65 |
| Tobramycin | 57 |
| Tenofovir | 48 |
| Cyclosporine | 13 |
| Naproxen | 8 |
| Amikacin | 7 |
| Diclofenac | 4 |
| Indomethacin | 3 |
| Polymyxin | 2 |
| Amphotericin | 2 |
| Cisplatin | 2 |
| Celecoxib | 2 |
| Methotrexate | 1 |
