# Supplementary material for: Prediction of Moderate-to-Severe Sepsis-Associated Acute Kidney Injury Using a Dual-Timepoint Machine Learning Model: Development, Multiregional Validation, and Clinical Deployment Study
Source: J Med Internet Res. 2025 Sep 30;27:e73840. doi: 10.2196/73840 (PMC12521856; doi:10.2196/73840)
Supplement: Multimedia Appendix 7 [file jmir_v27i1e73840_app7.docx]

| **Variables** | **48-Hour Group** | | | | **7-Day Group** | | | |
| --- | --- | --- | --- | --- | --- | --- | --- | --- |
|  | **AKI=Stages 0/1** | **AKI=Stages 2/3** | **Overall** | **P value** | **AKI=Stages 0/1** | **AKI=Stages 2/3** | **Overall** | **P value** |
|  | **(N=5560)** | **(N=7282)** | **(N=12842)** |  | **(N=3809)** | **(N=9033)** | **(N=12842)** |  |
| **Basic information** |  |  |  |  |  |  |  |  |
| Sex, n (%) |  |  |  |  |  |  |  |  |
| Male | 3197 (57.5) | 4140 (56.9) | 7337 (57.1) | 0.763 | 2190 (57.5) | 5147 (57.0) | 7337 (57.1) | 0.865 |
| Female | 2363 (42.5) | 3142 (43.1) | 5505 (42.9) |  | 1619 (42.5) | 3886 (43.0) | 5505 (42.9) |  |
| Age, years | 60.7 (17.0) | 64.6 (15.3) | 62.9 (16.2) | <.001 | 59.7 (17.3) | 64.2 (15.5) | 62.9 (16.2) | <.001 |
| **Interventions_24 hours, n (%)** |  |  |  |  |  |  |  |  |
| Ventilation | 3553 (63.9) | 6633 (91.1) | 10186 (79.3) | <.001 | 2063 (54.2) | 8123 (89.9) | 10186 (79.3) | <.001 |
| RRT^b^ | 28 (0.5) | 320 (4.4) | 348 (2.7) | <.001 | 18 (0.5) | 330 (3.7) | 348 (2.7) | <.001 |
| **Comorbidities, n (%)** |  |  |  |  |  |  |  |  |
| Arrhythmia | 1621 (29.2) | 2757 (37.9) | 4378 (34.1) | <.001 | 1028 (27.0) | 3350 (37.1) | 4378 (34.1) | <.001 |
| ARDS^c^, | 2155 (38.8) | 3707 (50.9) | 5862 (45.6) | <.001 | 1286 (33.8) | 4576 (50.7) | 5862 (45.6) | <.001 |
| Myocardial Infarction | 746 (13.4) | 1223 (16.8) | 1969 (15.3) | <.001 | 477 (12.5) | 1492 (16.5) | 1969 (15.3) | <.001 |
| Congestive Heart Failure | 1097 (19.7) | 1901 (26.1) | 2998 (23.3) | <.001 | 670 (17.6) | 2328 (25.8) | 2998 (23.3) | <.001 |
| Cerebrovascular Disease | 1149 (20.7) | 1217 (16.7) | 2366 (18.4) | <.001 | 740 (19.4) | 1626 (18.0) | 2366 (18.4) | 0.163 |
| Diabetes | 1207 (21.7) | 1993 (27.4) | 3200 (24.9) | <.001 | 777 (20.4) | 2423 (26.8) | 3200 (24.9) | <.001 |
| Severe Liver Disease | 228 (4.1) | 729 (10.0) | 957 (7.5) | <.001 | 125 (3.3) | 832 (9.2) | 957 (7.5) | <.001 |
| Malignant Cancer | 693 (12.5) | 986 (13.5) | 1679 (13.1) | 0.201 | 445 (11.7) | 1234 (13.7) | 1679 (13.1) | 0.009 |
| AIDS | 57 (1.0) | 40 (0.5) | 97 (0.8) | 0.00855 | 47 (1.2) | 50 (0.6) | 97 (0.8) | <.001 |
| **Vital signs (SD)** |  |  |  |  |  |  |  |  |
| Urine Output_24h, ml | 2510 (1350) | 1380 (962) | 1870 (1280) | <.001 | 2590 (1390) | 1560 (1090) | 1870 (1280) | <.001 |
| Temperature_max, °C | 37.7 (0.837) | 37.6 (0.848) | 37.6 (0.846) | <.001 | 37.7 (0.829) | 37.6 (0.850) | 37.6 (0.845) | <.001 |
| Heart Rate_max, bpm | 108 (20.5) | 109 (22.0) | 108 (21.3) | 0.902 | 108 (20.5) | 109 (21.7) | 108 (21.3) | 0.715 |
| Respiratory Rate_max, bpm | 28.5 (6.81) | 28.9 (6.84) | 28.7 (6.83) | <.001 | 28.3 (6.77) | 28.9 (6.86) | 28.7 (6.84) | <.001 |
| SBP_min, mmHg | 89.6 (15.9) | 86.6 (15.8) | 87.9 (15.9) | <.001 | 89.3 (15.6) | 87.4 (16.0) | 87.9 (15.9) | <.001 |
| DBP_min, mmHg | 46.8 (10.3) | 45.2 (10.5) | 45.9 (10.5) | <.001 | 46.5 (9.96) | 45.6 (10.6) | 45.9 (10.5) | <.001 |
| MBP_min, mmHg | 58.7 (13.2) | 56.8 (13.7) | 57.6 (13.5) | <.001 | 58.5 (12.8) | 57.2 (13.8) | 57.6 (13.5) | <.001 |
| **Laboratory tests (SD)** |  |  |  |  |  |  |  |  |
| pH | 7.36 (0.108) | 7.34 (0.117) | 7.34 (0.113) | <.001 | 7.36 (0.104) | 7.34 (0.115) | 7.34 (0.113) | <.001 |
| PaCO2, mmHg | 43.6 (12.9) | 44.9 (14.2) | 44.3 (13.7) | <.001 | 43.2 (12.7) | 44.8 (14.1) | 44.3 (13.7) | <.001 |
| OI^d^ | 299 (223) | 259 (202) | 276 (212) | <.001 | 317 (230) | 261 (201) | 277 (211) | <.001 |
| Base Excess | -1.33 (5.63) | -2.14 (6.12) | -1.79 (5.93) | <.001 | -1.22 (5.51) | -2.01 (6.08) | -1.78 (5.93) | <.001 |
| Potassium, mmol/L | 4.19 (0.810) | 4.31 (0.885) | 4.25 (0.855) | <.001 | 4.16 (0.807) | 4.29 (0.871) | 4.25 (0.855) | <.001 |
| Sodium, mmol/L | 138 (5.79) | 138 (5.92) | 138 (5.87) | 0.408 | 138 (5.73) | 138 (5.92) | 138 (5.87) | 0.043 |
| Chloride, mmol/L | 104 (7.20) | 103 (7.17) | 103 (7.19) | <.001 | 104 (7.19) | 103 (7.17) | 103 (7.19) | <.001 |
| Magnesium, mg/dL | 1.96 (0.458) | 1.98 (0.581) | 1.97 (0.531) | 0.317 | 1.96 (0.465) | 1.98 (0.558) | 1.97 (0.532) | 0.132 |
| Phosphate, mg/dL | 3.50 (1.33) | 3.82 (1.60) | 3.68 (1.50) | <.001 | 3.49 (1.34) | 3.77 (1.55) | 3.69 (1.50) | <.001 |
| Lactate, mmol/L | 2.25 (1.84) | 2.64 (2.64) | 2.48 (2.34) | <.001 | 2.16 (1.72) | 2.59 (2.53) | 2.47 (2.33) | <.001 |
| WBC, 10^3/uL | 13.8 (14.1) | 13.7 (9.57) | 13.8 (11.8) | 0.217 | 14.0 (15.4) | 13.7 (9.82) | 13.8 (11.8) | 0.419 |
| RBC, 10^6/uL | 3.77 (0.842) | 3.78 (0.878) | 3.77 (0.863) | 0.866 | 3.75 (0.847) | 3.78 (0.870) | 3.77 (0.863) | 0.193 |
| Platelets, 10^3/uL | 219 (118) | 214 (119) | 216 (119) | 0.009 | 218 (118) | 215 (119) | 216 (119) | 0.355 |
| Hemoglobin, g/dL | 11.3 (2.46) | 11.3 (2.55) | 11.3 (2.51) | 0.924 | 11.3 (2.48) | 11.3 (2.52) | 11.3 (2.51) | 0.83 |
| Hematocrit, | 34.2 (7.30) | 34.6 (7.63) | 34.4 (7.49) | 0.099 | 34.0 (7.32) | 34.6 (7.56) | 34.4 (7.49) | <.001 |
| BUN^e^, mg/dL | 22.4 (19.0) | 24.3 (18.9) | 23.5 (19.0) | <.001 | 21.9 (19.2) | 24.2 (18.8) | 23.5 (19.0) | <.001 |
| Creatinine, mg/dL | 1.09 (0.886) | 1.25 (1.09) | 1.18 (1.01) | <.001 | 1.09 (0.931) | 1.22 (1.04) | 1.18 (1.01) | <.001 |
| Total Bilirubin, mg/dL | 1.27 (2.69) | 1.87 (4.13) | 1.61 (3.59) | <.001 | 1.19 (2.19) | 1.81 (4.03) | 1.62 (3.60) | <.001 |
| AST, U/L | 188 (790) | 299 (1330) | 251 (1130) | <.001 | 197 (893) | 287 (1260) | 260 (1170) | <.001 |
| ALTe, U/L | 127 (516) | 174 (712) | 154 (635) | <.001 | 131 (578) | 172 (706) | 160 (671) | 0.004 |
| ALP, U/L | 105 (109) | 112 (126) | 109 (119) | <.001 | 101 (102) | 112 (125) | 109 (118) | <.001 |
| Glucose, mg/dL | 151 (92.9) | 158 (91.3) | 155 (92.0) | <.001 | 149 (97.4) | 157 (89.5) | 155 (92.0) | <.001 |
| Triglycerides, mg/dL | 202 (269) | 198 (268) | 200 (269) | 0.928 | 198 (254) | 193 (248) | 195 (250) | 0.833 |
| TyG^f^ | 9.18 (0.854) | 9.21 (0.862) | 9.20 (0.859) | 0.107 | 9.16 (0.843) | 9.21 (0.845) | 9.20 (0.844) | 0.003 |
| SHR^g^ | 1.20 (0.582) | 1.25 (0.565) | 1.23 (0.573) | <.001 | 1.17 (0.508) | 1.23 (0.536) | 1.21 (0.528) | <.001 |
| Albumin, g/dL | 3.24 (0.715) | 3.14 (0.718) | 3.18 (0.718) | <.001 | 3.23 (0.710) | 3.15 (0.715) | 3.18 (0.715) | <.001 |
| PT, seconds | 15.6 (8.70) | 17.1 (11.0) | 16.4 (10.1) | <.001 | 15.4 (7.96) | 16.8 (10.7) | 16.4 (10.0) | <.001 |
| INR^h^ | 1.42 (0.832) | 1.56 (1.04) | 1.50 (0.959) | <.001 | 1.40 (0.778) | 1.54 (1.02) | 1.50 (0.954) | <.001 |
| PTT, seconds | 34.6 (19.4) | 37.7 (23.3) | 36.4 (21.7) | <.001 | 34.3 (18.7) | 37.3 (22.9) | 36.4 (21.8) | <.001 |
| Fibrinogen, mg/dL | 332 (185) | 337 (188) | 335 (187) | 0.277 | 324 (182) | 339 (189) | 335 (188) | <.001 |
| Anion Gap, mmol/L | 14.9 (4.72) | 15.4 (5.19) | 15.2 (5.00) | <.001 | 14.9 (4.71) | 15.3 (5.11) | 15.2 (5.00) | <.001 |
| RDW^i^, | 14.6 (2.13) | 15.1 (2.41) | 14.8 (2.31) | <.001 | 14.5 (2.07) | 15.0 (2.39) | 14.8 (2.31) | <.001 |
| **Drug use, 24 hr** |  |  |  |  |  |  |  |  |
| Nephrotoxic Drugs, n (%) | 1421 (37.3) | 5570 (61.7) | 6991 (54.4) | <.001 | 2399 (43.1) | 4592 (63.1) | 6991 (54.4) | <.001 |
| Nephrotoxic Score (SD) | 0.571 (0.739) | 0.850 (0.784) | 0.729 (0.777) | <.001 | 0.505 (0.730) | 0.824 (0.777) | 0.729 (0.777) | <.001 |
| Epinephrine Hours (SD) | 0.221 (1.88) | 0.382 (2.41) | 0.312 (2.20) | <.001 | 0.238 (1.97) | 0.343 (2.28) | 0.312 (2.20) | <.001 |
| Norepinephrine Hours (SD) | 2.35 (6.20) | 4.60 (8.14) | 3.63 (7.44) | <.001 | 1.83 (5.50) | 4.38 (8.00) | 3.63 (7.44) | <.001 |
| Vasopressin Hours (SD) | 0.485 (2.83) | 1.41 (4.72) | 1.01 (4.04) | <.001 | 0.354 (2.40) | 1.28 (4.53) | 1.01 (4.04) | <.001 |
| **Score (SD)** |  |  |  |  |  |  |  |  |
| ^j^SOFA | 5.12 (3.02) | 6.51 (3.81) | 5.91 (3.56) | <.001 | 5.00 (2.90) | 6.29 (3.73) | 5.91 (3.56) | <.001 |
| ^k^GCS | 13.1 (3.27) | 13.2 (3.30) | 13.1 (3.29) | <.001 | 13.1 (3.21) | 13.1 (3.33) | 13.1 (3.29) | 0.057 |

^a^ SA-AKI: sepsis-associated acute kidney injury.

^b^ ARDS: acute respiratory distress syndrome.

^c^ RRT: Renal Replacement Therapy.

^d^ OI, Oxygenation Index.

^e^ BUN: blood urea nitrogen.

^f^ TyG Index, Triglyceride-Glucose Index.

^g^ SHR, Stress Hyperglycemia Ratio.

^h^ INR: international normalized ratio.

^i^ RDW, Red Cell Distribution Width.

^j^ SOFA, Sequential Organ Failure Assessment.

^k^ GCS, Glasgow Coma Scale.
