# Supplementary material for: Prediction of Moderate-to-Severe Sepsis-Associated Acute Kidney Injury Using a Dual-Timepoint Machine Learning Model: Development, Multiregional Validation, and Clinical Deployment Study
Source: J Med Internet Res. 2025 Sep 30;27:e73840. doi: 10.2196/73840 (PMC12521856; doi:10.2196/73840)
Supplement: Multimedia Appendix 10 [file jmir_v27i1e73840_app10.docx]

| **Model Framework** | **48Hour AUC^a^  (95% CI^b^)** | **48Hour  *P*-value^c^** | **48Hour F1 Score  (95% CI)** | **7Day AUC  (95% CI)** | **7Day  *P*-value^c^** | **7Day F1 Score  (95% CI)** |
| --- | --- | --- | --- | --- | --- | --- |
| Dual Independent Model | 0.839 (0.824-0.854) | Ref | 0.755 (0.738-0.771) | 0.834 (0.818-0.850) | Ref | 0.734 (0.714-0.754) |
| 48h-Specific Model | 0.839 (0.824-0.854) | 1.00 | 0.755 (0.738-0.771) | 0.835 (0.819-0.852) | .910 | 0.692 (0.673-0.710) |
| 7d-Specific Model | 0.830 (0.814-0.846) | .165 | 0.662 (0.643-0.682) | 0.834 (0.818-0.850) | 1.00 | 0.734 (0.714-0.754) |
| Union 16-Feature 48h Model^d^ | 0.838 (0.822-0.853) | .832 | 0.747 (0.730-0.764) | 0.833 (0.817-0.849) | .896 | 0.690 (0.673-0.709) |
| Union 16-Feature 7d Model^e^ | 0.833 (0.817-0.850) | .298 | 0.734 (0.713-0.752) | 0.831 (0.815-0.846) | .663 | 0.670 (0.651-0.689) |
| Unified Union Feature Model^f^ | 0.835 (0.820-0.851) | .622 | 0.745 (0.728-0.762) | 0.833 (0.817-0.849) | .907 | 0.729 (0.708-0.747) |

^a^ AUC: area under the curve.
^b^ CI: confidence interval.
^c^ P-values: Calculated using the DeLong test, comparing each model framework against the dual independent model (reference).
^d^ Union 16-Feature 48h Model: Model using the combined union feature set (16 features from both timepoints) trained specifically for 48-hour SA-AKI prediction.
^e^ Union 16-Feature 7d Model: Model using the combined union feature set (16 features from both timepoints) trained specifically for 7-day SA-AKI prediction.
^f^ Unified Union Feature Model: Multi-task model using the combined union feature set (16 features) with shared architecture to simultaneously predict both 48-hour and 7-day SA-AKI outcomes.
